# Forecasting and mapping dengue fever epidemics in China: a spatiotemporal analysis

**DOI:** 10.1186/s40249-024-01219-y

**Published:** 2024-07-03

**Authors:** Hongyan Ren, Nankang Xu

**Affiliations:** 1 State Key Laboratory of Resources and Environmental Information System, Institute of Geographic Sciences and Natural Resources Research, Chinese Academy of Sciences, Beijing, 100101 China; 2 College of Resources and Environment, University of Chinese Academy of Sciences, Beijing, 100049 China

**Keywords:** Dengue fever, Imported case, Inevitability, Occasionality, Time window, Random forest, China

## Abstract

**Background:**

Dengue fever (DF) has emerged as a significant public health concern in China. The spatiotemporal patterns and underlying influencing its spread, however, remain elusive. This study aims to identify the factors driving these variations and to assess the city-level risk of DF epidemics in China.

**Methods:**

We analyzed the frequency, intensity, and distribution of DF cases in China from 2003 to 2022 and evaluated 11 natural and socioeconomic factors as potential drivers. Using the random forest (RF) model, we assessed the contributions of these factors to local DF epidemics and predicted the corresponding city-level risk.

**Results:**

Between 2003 and 2022, there was a notable correlation between local and imported DF epidemics in case numbers (*r* = 0.41, *P* < 0.01) and affected cities (*r* = 0.79, *P* < 0.01). With the increase in the frequency and intensity of imported epidemics, local epidemics have become more severe. Their occurrence has increased from five to eight months per year, with case numbers spanning from 14 to 6641 per month. The spatial distribution of city-level DF epidemics aligns with the geographical divisions defined by the Huhuanyong Line (Hu Line) and Qin Mountain-Huai River Line (Q-H Line) and matched well with the city-level time windows for either mosquito vector activity (83.59%) or DF transmission (95.74%). The RF models achieved a high performance (AUC = 0.92) when considering the time windows. Importantly, they identified imported cases as the primary influencing factor, contributing significantly (24.82%) to local DF epidemics at the city level in the eastern region of the Hu Line (E–H region). Moreover, imported cases were found to have a linear promoting impact on local epidemics, while five climatic and six socioeconomic factors exhibited nonlinear effects (promoting or inhibiting) with varying inflection values. Additionally, this model demonstrated outstanding accuracy (hitting ratio = 95.56%) in predicting the city-level risks of local epidemics in China.

**Conclusions:**

China is experiencing an increasing occurrence of sporadic local DF epidemics driven by an unavoidably higher frequency and intensity of imported DF epidemics. This research offers valuable insights for health authorities to strengthen their intervention capabilities against this disease.

**Graphical Abstract:**

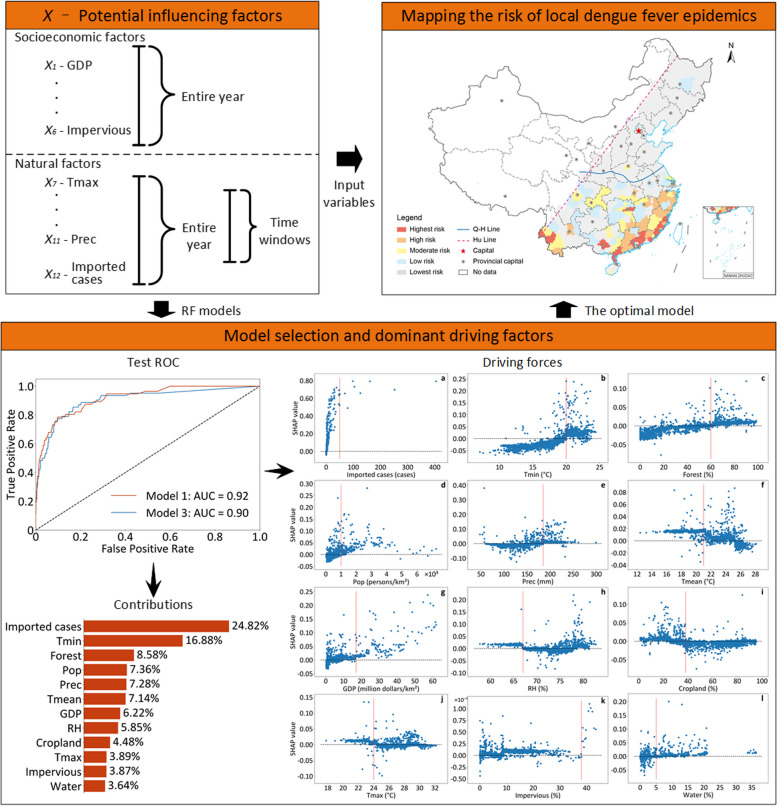

**Supplementary Information:**

The online version contains supplementary material available at 10.1186/s40249-024-01219-y.

## Background

Dengue fever (DF) is an acute infectious disease caused by the dengue virus and is transmitted by *Aedes albopictus* and *Aedes aegypti* [1]. DF is naturally widely distributed in tropical and subtropical regions worldwide (e.g., Southeast Asia, the Western Pacific, and South Africa) [2], causing approximately one-third of the global population to be exposed to this disease [3]. Due to the impact of globalization and climate change in recent years, the increasing incidence and range of DF epidemics have become significant public health concerns.

Epidemics of DF in China are mainly caused by imported cases from natural foci around the world. No DF cases were reported between 1949 and 1978; however, China has experienced an intermittent DF epidemic since the sudden outbreak in Foshan, Guangdong Province in 1978 [4]. Since the 1990s, DF has been included in the list of notifiable communicable diseases [5]. In recent years, local DF outbreaks have increased substantially due to an increase in imported cases caused by the increasing severity of global DF epidemics and China’s growing significance in international trade and economy [6, 7]. Moreover, DF epidemics have expanded geographically from southern China (southwest border and southeast coastal areas) toward northern inland regions, such as Jiangxi (Yichun), Chongqing, Henan (Xuchang), and Shandong (Jining) [8, 9]. Overall, the DF epidemic in China has shown increasingly shorter time intervals and apparent spatial expansion.

Numerous studies have investigated the factors that affect the spread and prevalence of DF at various levels, including dengue virus, mosquito vectors, susceptible populations, environmental conditions, and socioeconomic status [10, 11]. Among these influencing factors, environmental conditions, such as climate, hydrology, and vegetation coverage, primarily affect the activity of the dengue virus and the breeding, survival, surroundings, and biting behavior of mosquito vectors [12, 13]. Socioeconomic factors, such as population density and mobility, land use, accessibility of public transportation, residents’ income level, and living habits, chiefly influence the probability of human-mosquito contact [14, 15]. In general, the transmission and prevalence of DF are complex and involve intricate geo-ecological processes.

Previous studies on the spatial and temporal variations in DF epidemics at various scales and their influencing factors have strongly supported DF prevention and control in the absence of effective clinical vaccines and improved our understanding of the prevalence and spread of the disease in China [16, 17]. However, questions remain regarding the future of China’s DF epidemic, especially in the absence of an effective vaccine and the presence of inextirpable DF foci worldwide. First, is a DF epidemic unavoidable in China in the future? Second, what factors control the spatial expansion of local epidemics? Finally, how can we reliably predict the risk of local DF epidemics?

Thus, we conducted this study to 1) characterize the spatiotemporal situation of China’s DF epidemic at the city level, 2) quantify the contributions of various environmental and socioeconomic factors to the occurrence of local DF epidemics via random forest (RF) modeling, and 3) predict the city-level risk of local DF epidemics in China.

## Methods

### Data on dengue cases

Cases of DF in China were sourced from the National Infectious Disease Surveillance System from 2003 to 2022. The dataset contains information about each patient's date of onset, residential address, and reporting address. The cases were classified as imported or local according to their origin. Imported cases were those occurring in individuals who had traveled to a dengue-endemic country or region within 14 days before the onset of the disease. The number of imported cases was preliminarily considered an important influencing factor since it constantly triggered local DF transmission in China. Local cases were those occurring in individuals who had not left the city within 14 days before the onset of the disease or those who had left the city within 14 days before the onset of the disease and visited other domestic dengue epidemic cities [18]. An epidemic outbreak was defined as the occurrence of three or more local cases within the maximum incubation period (14 days) in a city. If no local cases were reported within 14 days after the outbreak, the outbreak was considered to have ended. The duration from the beginning of a local DF outbreak to its end was referred to as the outbreak period. After filtering isolated local cases occurring outside the outbreak period, imported and local cases were aggregated at the city level according to the address codes of the cases.

### Potential influencing factors

According to previous studies [10–15], we identified 11 potential influencing factors, including six socioeconomic and five meteorological factors (Table 1). The mean values of gross domestic product (GDP) and population density (Pop) within each city were retrieved from yearly 1 km × 1 km raster data; percentages of four land-use types (cropland, forest, water, and impervious) were extracted from yearly 30 m × 30 m gridded data for each city during 2003–2022. Moreover, the monthly average values of each meteorological element within each city, such as the maximum temperature (Tmax), minimum temperature (Tmin), average temperature (Tmean), average relative humidity (RH), and precipitation (Prec), were extracted from monthly 1 km × 1 km gridded climatic data. The annual mean values of these five climatic elements were further calculated based on the monthly average values at the city level. These data were processed using zonal statistics and spatial connectivity tools in ArcGIS 10.6 (ESRI, Redlands, CA, USA). If data were missing for certain years, data of an adjacent year were used as a replacement.

**Table 1:** Data collection and sources used in this study

Data group	Selected variables from previous studies	Data unit	Time scale	Resolution	Source
	Number of imported cases (Imported cases)	Cases	Annual	City level	The National Infectious Disease Surveillance System
Socioeconomic factors	Gross domestic product per capita (GDP) [10, 14]	Million dollars/km^2^	Annual	1 km	The Scientific Data (https://doi.org/10.6084/m9.figshare.17004523.v1) The WorldPop (https://hub.worldpop.org/) Earth System Science Data (https://doi.org/10.5281/zenodo.5816591)
	Population density (Pop) [10, 14, 15]	Persons/km^2^	Annual	1 km	
	Annual average percentage of cropland (Cropland) [15]	%	Annual	30 m	
	Annual average percentage of forest (Forest) [15]	%	Annual	30 m	
	Annual average percentage of water (Water) [15]	%	Annual	30 m	
	Annual average percentage of impervious (Impervious) [15]	%	Annual	30 m	
Natural factors	Average monthly maximum air temperature (Tmax) [11, 12]	Degree Celsius	Monthly	1 km	Earth System Science Data (https://doi.org/10.5281/zenodo.5112232) National Earth System Science Data Center (http://www.geodata.cn) National Tibetan Plateau/Third Pole Environment Data Center (https://data.tpdc.ac.cn/)
	Average monthly mean air temperature (Tmean) [11–13]	Degree Celsius	Monthly	1 km	
	Average monthly minimum air temperature (Tmin) [11, 12]	Degree Celsius	Monthly	1 km	
	Average monthly mean relative humidity (RH) [13]	%	Monthly	1 km	
	Average monthly precipitation (Prec) [11–13]	0.1 mm	Monthly	1 km	

As illustrated in Additional file 1: (Fig. S2), the above 11 city-level variables presented obvious spatial differences around two famous geographical dividing lines, namely, the Huhuanyong Line (Hu Line) and Qin Mountain-Huai River Line (Q-H Line) [19, 20]. For example, the city-level population density in the eastern region of the Hu Line (E–H region) was much greater than that in the western region of the Hu Line (W–H region). Moreover, two subregions around the Q-H Line, the northern region (N-QH region) and the southern region (S-QH region), were identified due to their marked differences in meteorological conditions.

### Descriptive analysis of the frequency and intensity of DF epidemics in China

The numbers of local and imported cases were aggregated annually from 2003 to 2022, and the relationships between local and imported cases were analyzed using Spearman correlation analysis. The cities with imported or local epidemics were categorized into four groups: cities experiencing either imported or local epidemics, cities experiencing both types of epidemics, cities experiencing only imported epidemics, and cities experiencing only local epidemics. Moreover, we explored the spatial distribution characteristics of these cities in different groups, and investigated the Spearman correlation coefficients between the numbers of cities experiencing local epidemics and the numbers of cities experiencing imported epidemics each year across China. In addition, two indicators, namely, the frequency and intensity of DF epidemics, were established to characterize their spatial and temporal variations at the country and city levels (Table 2), providing a comprehensive understanding of China’s DF epidemics.

**Table 2:** Definition of the frequency and intensity of dengue fever epidemics

Indicators	Spatial unit	Definition
Frequency	City	Annual number of months experiencing dengue fever epidemics
		Annual number of local dengue fever outbreaks
	Countrywide	Annual number of months experiencing dengue fever epidemics
		Annual number of local dengue fever outbreaks
Intensity	City	Average number of cases in the months experiencing dengue fever epidemics
		Average number of cases per outbreak
	Countrywide	Average number of cases in the months experiencing dengue fever epidemics
		Average number of cases per outbreak

### Retrieval of time windows

The suitability of environmental conditions, including temperature, humidity, and precipitation, is a crucial determinant of mosquito breeding and activities, which in turn affects the spread of local epidemics. Previous studies have shown that monthly minimum temperatures exceeding 10°C [21–23], monthly average temperatures between 15°C and 32°C [24, 25], monthly maximum temperatures below 38°C [22, 23], monthly average relative humidity between 60% and 90% [26, 27], and monthly precipitation between 60 mm and 650 mm [28, 29] are conducive to *Aedes* mosquito breeding. Accordingly, we defined the periods during which all these conditions were met as the time windows for mosquito vector activity. To account for the incubation periods of dengue viruses [30], we postponed the ending time of the time windows for mosquito vector activity by one month while keeping the beginning time unchanged and accepted them as the time windows for local DF transmission. To evaluate the effectiveness of the time windows, we calculated the match degree using the following formula:

$$D=n/N\times 100\%$$

where $D$ is the match degree, $n$ refers to the number of local DF outbreaks that occurred within the time windows, and $N$ refers to the total number of local DF outbreaks. A higher match degree indicates a greater proportion of local DF outbreaks occurring within the time window.

### Identification of the driving forces of local DF epidemics at the city level

We transformed the number of local cases in a year into a binary variable (0 = absence and 1 = presence or occurrence) and used it as the dependent variable, whereas the number of imported cases within the time windows was used as an independent variable at the city level. Moreover, we considered six socioeconomic factors and five meteorological elements (i.e., their average values in the months within the time windows). The correlation coefficients between the occurrence of local DF epidemics and the input variables were determined using the Spearman method before identifying the driving forces of local DF epidemics.

The RF, gradient boosting machine (GBM), and support vector machine (SVM) methods are powerful machine learning methods for classification and regression [31–33] and are typically employed to fit the relationships between local epidemics and their influencing factors and identify the driving forces of DF. In this study, the data from 2003 to 2018 were divided into a training set (70%) and a test set (30%), while the data from 2019 to 2022 were used as the prediction set. In the RF model, the number of trees to grow was the main parameter, and we used a range of tree numbers (from 100 to 2000, step = 100) to select the optimal parameter according to model performance. In the GBM model, the number of trees to grow and the learning rate were the main parameters, and we used a range of tree numbers (from 100 to 2000, step = 100) and learning rates (from 0.01 to 0.2, step = 0.01). In the SVM model, we applied “*rbf*” as the kernel function and used a range of regularization parameters (from 0.1 to 3, step = 0.1) and kernel coefficients (from 0.01 to 0.5, step = 0.01). We performed fivefold cross-validation on all three models to increase the modeling stability. The area under the curve (AUC) of the receiver operating characteristic (ROC) curve was used to measure the model’s predictive ability [34]. In this study, the AUC values were classified as follows: 0.50–0.70, indicating a poor model; 0.70–0.80, suggesting an average model; and 0.80–1.00, reflecting a good model [35, 36]. We selected an optimal model from the above three models. In addition, to analyze the impact of the time windows, we refitted the models without considering the time windows and instead considered the number of imported cases and the average values of meteorological factors throughout the year.

As a valuable parameter of model evaluation, the SHapley Additive exPlanations (SHAP) values (i.e., global and local values) are widely utilized to interpret the results derived from various machine learning models [37–39]. We employed SHAP values to quantify the contributions of various factors and driving forces to local epidemics. A direct relationship was observed between the absolute value of a variable’s global SHAP and its contribution to the model; i.e., a larger absolute value of a variable’s global SHAP corresponded to a greater contribution by that variable to the model. In contrast, the local SHAP values of each variable represent its driving force on local epidemics. Positive SHAP values indicate the promoting effects of the variable, with larger values representing stronger promoting influences. In contrast, negative SHAP values indicate the inhibiting effects of the variable, with smaller values indicating stronger inhibiting effects. In this study, we utilized the *sklearn* and *shap* packages in Python 3.7.4 (Python Software Foundation, Delaware, USA) to construct and interpret the models.

### Predicting the occurrence probability of local DF epidemics at the city level

The optimal models were built and evaluated using the AUC values considering the time windows for local DF transmission and utilized to predict the probability of city-level occurrence of local DF epidemics in the E–H region from 2019 to 2022. Then, the city-level probability was classified into five levels to indicate the risk of local DF epidemics in each city, ranging from the highest to the lowest: 0.80–1, 0.60–0.80, 0.40–0.60, 0.20–0.40, and 0–0.20. Moreover, the accuracy of these prediction models was assessed using the hitting ratios in terms of the percentages of cities with actual local DF epidemics from 2019 to 2022 in those identified by the models. A direct relationship was observed between the hitting ratio of the prediction model and the capacity of the model to predict risk of local DF epidemics at the city level; i.e., a higher hitting ratio of a prediction model corresponded to a stronger capacity to predict the risk of local DF epidemics at the city level.

## Results

### Current situation of DF epidemics in China

**Fig. 1:**
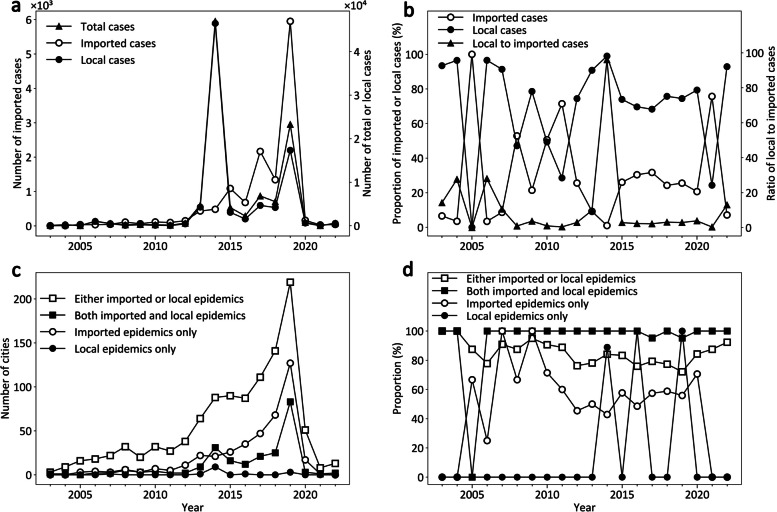
Temporal variations in dengue fever epidemics in China during 2003–2022. **a** Yearly numbers of total, imported, and local DF cases; **b** Yearly proportions of imported and local DF cases to total cases, and the ratio of local cases to imported cases; **c** Yearly numbers of cities with different DF epidemics; **d** Yearly proportions of cities with different DF epidemics in the S-QH region. *DF* Dengue fever; *S-QH region* The southern region of the Q-H Line

From 2003 to 2022, a total of 329 DF outbreaks occurred within 104 months with 98,560 DF cases in China; the number of DF outbreaks displayed an overall upward trend, while the numbers of both imported and local DF cases sharply decreased from 2020 to 2022 (Fig. 1a). The proportions of local DF cases were much greater than those of imported cases over 15 years, especially since 2012 (Fig. 1b). At the city level, the numbers of local DF cases in China were increasingly closely associated with those of imported cases from 2003 to 2012 (*r* = 0.23, *P* < 0.01), 2013 to 2022 (*r* = 0.44, *P* < 0.01), and 2003 to 2022 (*r* = 0.41, *P* < 0.01).

Moreover, an increasing number of cities are affected by DF epidemics. Despite an acute decrease in DF between 2020 and 2022, the numbers of cities with imported or local epidemics, both types of epidemics, or only imported epidemics displayed similar temporal variations (Fig. 1c). In contrast, the number of cities with only local epidemics remained below 3, except for 8 cities in 2014 (Fig. 1c). Nevertheless, the number of cities experiencing local epidemics was significantly associated with the number of cities experiencing imported epidemics at the national level (*r* = 0.79, *P* < 0.01).

**Fig. 2:**
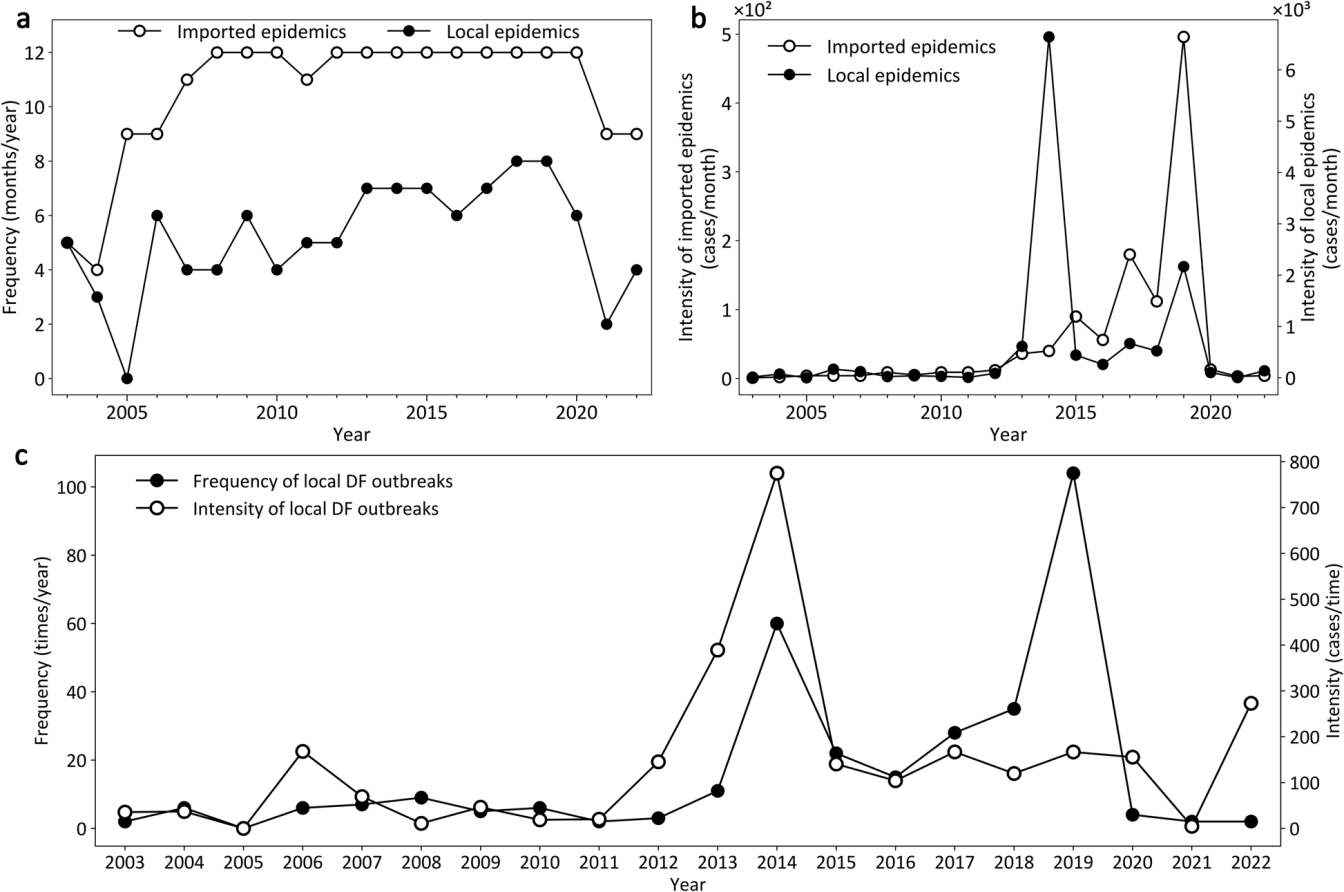
Temporal variations in the frequency and intensity of dengue fever epidemics in China from 2003 to 2022. **a** The frequencies of imported DF epidemics and local DF epidemics; **b** The intensities of imported DF epidemics and local DF epidemics; **c** Temporal variations in the frequency and intensity of local DF outbreaks. *DF* Dengue fever

China’s DF epidemic also displayed obvious temporal variations in frequency and intensity. As illustrated in Fig. 2a, the frequency of imported DF epidemics was less than 9 months per year before 2006 and then increased rapidly to 11 months per year or more since 2007, with a decrease to 9 months in 2021–2022. In comparison, the frequency of local epidemics exhibited a relatively slow and fluctuating uptrend, varying from 0 to 8. However, in terms of intensity, local DF epidemics were more severe (from 14 to 6641 cases per month) than imported DF epidemics (from one to 496 cases per month), despite the latter displaying a steadier and quicker increase (Fig. 2b). In addition, another frequency (i.e., the times of local DF outbreaks per year) presented a clear uptrend, and its intensity (i.e., the number of local DF cases per time) has remained higher than 100 cases per time since 2012 (Fig. 2c). These analyses showed that local DF epidemics in China have become increasingly severe over time as the number of imported DF cases has increased.

### Geographical distribution of the DF epidemic

The distributions of the cities with DF epidemics were spatially featured across China. As illustrated in Additional file 1: (Fig. S4), the vast majority (96–100%) of the cities with imported or local epidemics were distributed in the E–H region. Among them, more than 75% were located in the S-QH region and displayed a downward trend from 2012 (Fig. 1d), implying that China’s DF epidemics were expanding toward the N-QH region. Moreover, the overwhelming majority of the cities with both imported and local epidemics were located in the S-QH region (Fig. 1d). In comparison, most of the cities with only imported epidemics were located in the E–H region, while those with only local epidemics were much fewer.

**Fig. 3:**
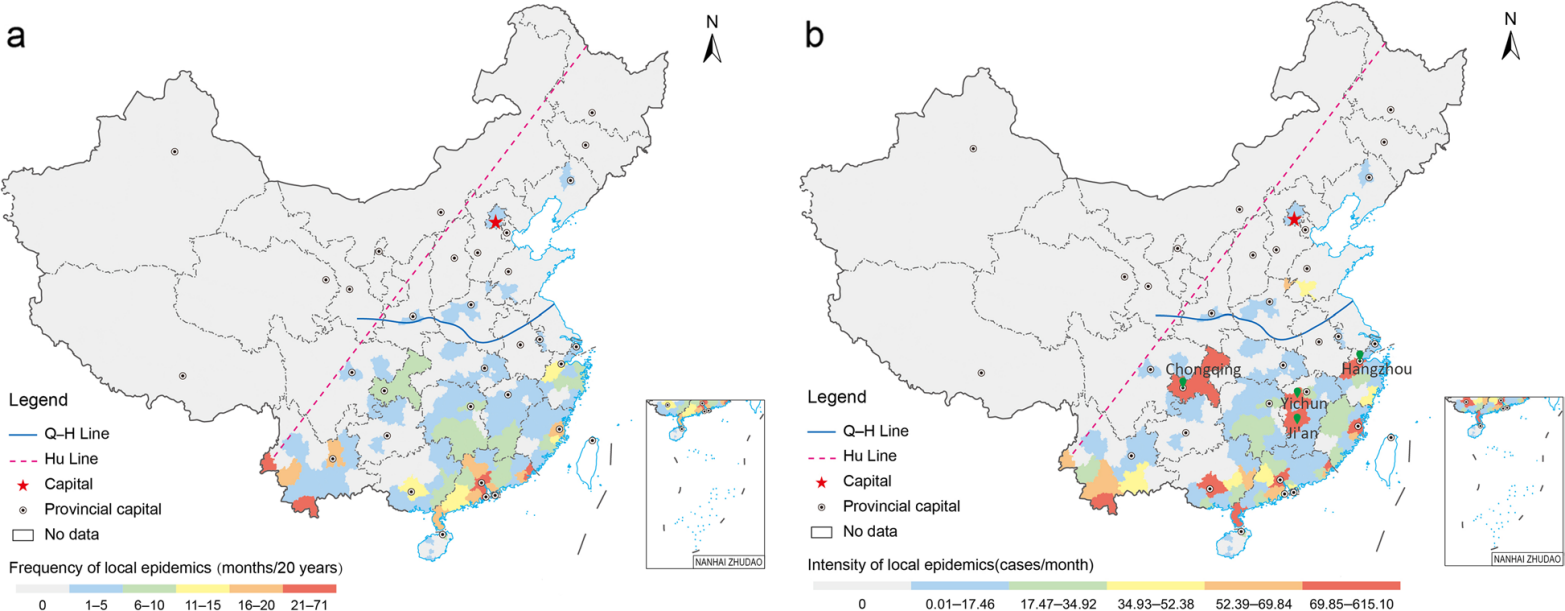
The distribution of the frequency and intensity of local dengue fever epidemics at the city level during 2003–2022. **a** The frequency; **b** The intensity. *Hu Line* The Huhuanyong Line; *Q-H Line* The Qin Mountain-Huai River Line. Map approval No.: GS (2024)2127

Moreover, the frequency and intensity of imported and local DF epidemics presented geographical disparities at the city level. The cities with an average frequency of imported epidemics greater than one month per year were mainly distributed in the E–H region (Additional file 1: Fig. S5a). A few cities with an average frequency much greater than 1.5 months per year were sparsely located, especially in the S-QH region. In contrast, fewer cities had an average frequency of local epidemics greater than 0.5 months per year and were mainly concentrated in the S-QH region (Fig. 3a). Similarly, the cities with a relatively high intensity of imported epidemics were mainly located in the S-QH region (Additional file 1: Fig. S5b), whereas those with a relatively high intensity of local epidemics were sparsely distributed in this region (Fig. 3b). Notably, provincial capitals often had a much higher frequency and intensity of imported epidemics, and some inland cities, such as Ji’an, Yichun, Chongqing, and Hangzhou, had the highest intensity of local epidemics despite a relatively lower frequency (Fig. 3b).

### Time windows for local DF epidemics

**Fig. 4:**
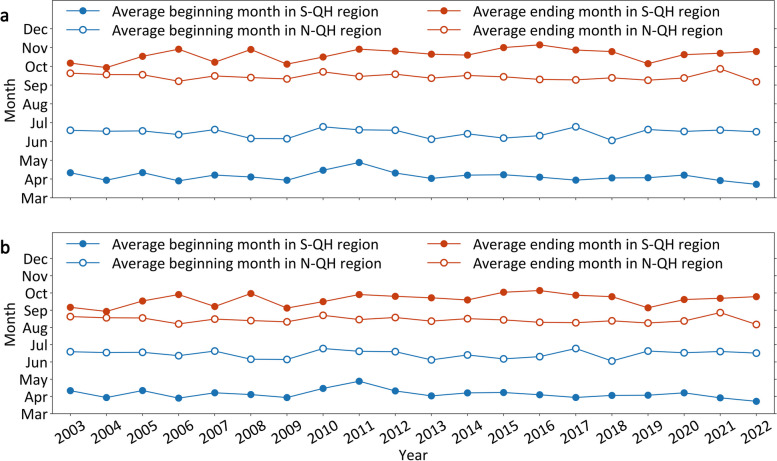
The durations from the beginning to the ending months of time windows in the S-QH and N-QH regions during 2003–2022. **a** Average beginning and ending months of time windows for local DF transmission; (**b**) Average beginning and ending months of time windows for mosquito vector activity. *DF* Dengue fever; *S-QH region* The southern region of the Q-H Line; *N-QH region* The northern region of the Q-H Line; *Hu Line* The Huhuanyong Line; *Q-H Line* The Qin Mountain-Huai River Line

The city-level time windows for local DF transmission exhibited obvious spatial differences on either side of the Hu Line and Q-H Line (Additional file 1: Fig. S7). No time windows were observed in most of the cities in the W–H region throughout the year, whereas the cities in the E–H region presented various durations of time windows for local DF transmission. Furthermore, the city-level time windows in the E–H region exhibited significant geographical variation when compared to the S-QH and N-QH regions because of differences in their beginning and ending months as well as durations (Fig. 4a). In the N-QH region, the time windows often opened relatively later (beginning in June–July) and closed earlier (ending in September–October), and a few cities occasionally closed their time windows in some years (e.g., 2003–2004, 2006–2007, 2009–2011, and 2014–2016). In contrast, the time windows in the S-QH region began earlier (April–May) and ended later (October–November). As a result, these two regions exhibited long (seven or eight months in the S-QH region) or short (three or four months in the N-QH region) durations of time windows. Similarly, the city-level time windows for mosquito vector activity were also obviously spatially differentiated between the S-QH and N-QH regions due to the variations in their durations (Fig. 4b). Regarding the match degree, the time windows for local DF transmission were much greater (95.74%) than those for mosquito vector activity (83.59%), although the time windows for mosquito vector activity displayed similar spatial differences at the city level (Additional file 1: Fig. S6).

### Analysis of the relationships among the time windows

Within the time windows for local DF transmission in the E–H region, the occurrence of local DF epidemics showed a significant positive correlation with imported cases (*r* = 0.42, *P* < 0.01) and most of the natural and socioeconomic factors (Table 3), except for Cropland (*r* = -0.14, *P* < 0.01). In contrast, the relationships observed in the S-QH and N-QH regions differed. Local DF epidemics were significantly negatively associated with Forest (*r* = -0.09) in the S-Q region and positively associated with only three factors in the N-Q region, namely, imported cases (*r* = 0.12), GDP (*r* = 0.08), and Pop (*r* = 0.08), at a significance level of 0.01.

**Table 3:** Correlation coefficients between the occurrence of local dengue fever epidemics and potential influencing factors

Data group	Variable name	Correlation coefficients		
		The E–H region	The N-QH region	The S-QH region
Socioeconomic factors	Imported cases	0.42^a^	0.12^a^	0.45^a^
	GDP	0.17^a^	0.08^a^	0.25^a^
	Pop	0.14^a^	0.08^a^	0.18^a^
	Cropland	-0.14^a^	0.01	-0.09^a^
	Forest	0.10^a^	-0.01	0.01
	Water	0.13^a^	0.02	0.11^a^
	Impervious	0.06^a^	0.06^b^	0.18^a^
Natural factors	Tmax	0.09^a^	0.02	0.11^a^
	Tmean	0.13^a^	0.02	0.11^a^
	Tmin	0.16^a^	0.02	0.12^a^
	RH	0.14^a^	-0.03	0.07^a^
	Prec	0.21^a^	-0.03	0.18^a^

*Imported cases* Number of imported cases, *GDP* Gross domestic product, *Pop* Population density, *Cropland* Annual average percentage of cropland, *Forest* Annual average percentage of forest, *Water* Annual average percentage of water, *Impervious* Annual average percentage of impervious, *Tmax* Average monthly maximum air temperature, *Tmean* Average monthly mean air temperature, *Tmin* Average monthly minimum air temperature, *RH* Average monthly mean relative humidity, *Prec* Average monthly precipitation, *E–H region* The eastern region of the Hu Line, *N-QH region* The northern region of the Q-H Line, *S-QH region* The southern region of the Q-H Line

^a^ and ^b^ indicates this value is significant at the level of 0.01 and 0.05

Furthermore, RF models were utilized to explore the relationships between the occurrence of local DF epidemics and influencing factors at the city level because of their superior ability beyond that of the GBM and SVM models (Additional file 1: Table S6). According to the higher AUC values in Table 4, the RF models in the E–H region (Models 1, 2, and 3) performed considerably better than the other models, although Model 4 also showed good performance in the S-QH region (AUC = 0.85). Moreover, the AUC slightly increased from 0.90 (Model 3) to 0.92 (Models 1 and 2) when considering the time windows for either local DF transmission or mosquito vector activity in the E–H region. These results showed that RF models considering time windows could be rationally applied to explore the contributions of factors and predict the city-level risk of local DF epidemics.

**Table 4:** The AUC values derived from the random forest models

		**Training**	**Testing**	**Prediction**
The E–H region	Model 1	0.87	0.92	0.91
	Model 2	0.87	0.92	–
	Model 3	0.89	0.90	–
The S-QH region	Model 4	0.88	0.85	–
	Model 5	0.86	0.86	–
	Model 6	0.85	0.91	–

Model 1 and Model 4 respectively represented the models considering the time windows for local DF transmission in the E–H region and the S-QH region

Model 2 and Model 5 respectively represented the models considering the time windows for mosquito vector activity in the E–H region and the S-QH region

Model 3 and Model 6 respectively represented the models without regard for the time windows in the E–H region and the S-QH region

*DF* Dengue fever, *E–H region* The eastern region of the Hu Line, *S-QH region* The southern region of the Q-H Line, *AUC* Area Under the Curve

–Not applicable

### Dominant influencing factors of local DF epidemics

**Fig. 5:**
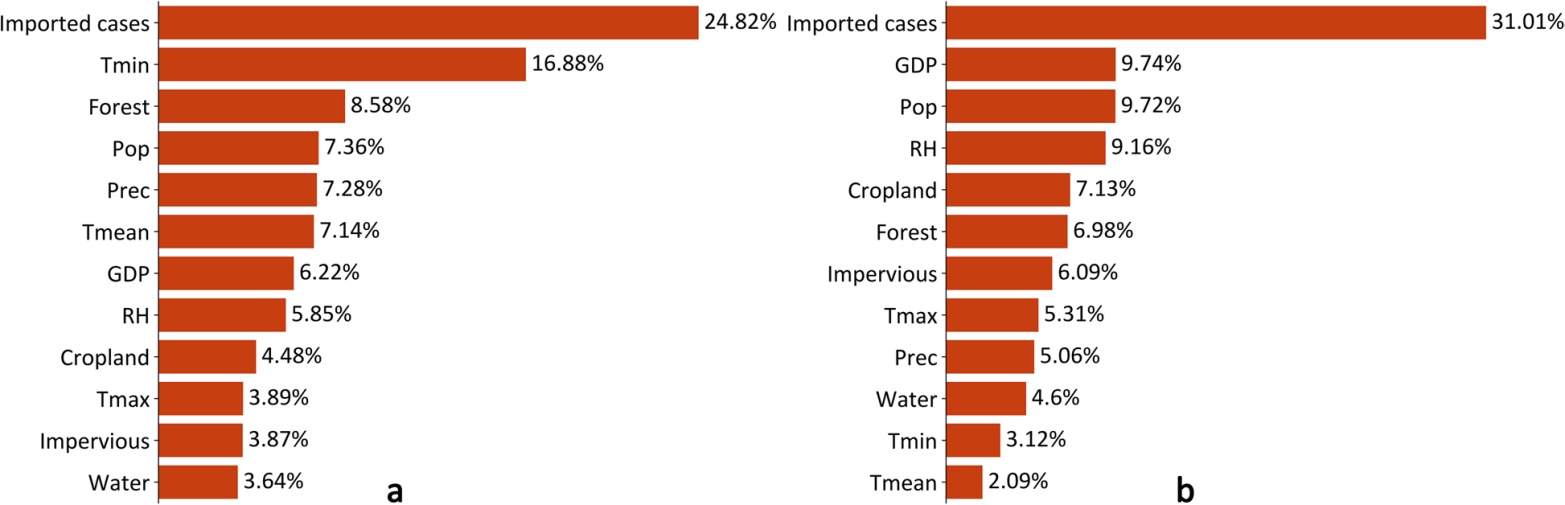
Contributions of the input variables based on the global SHAP values in the model considering the time windows for local dengue fever transmission. **a** The contributions of input variables in the E–H region; **b** The contributions of input variables in the S-QH region. *Imported cases* Number of imported cases; *Tmin* Average monthly minimum air temperature; *Forest* Annual average percentage of forest; *Pop* Population density; *Prec* Average monthly precipitation; *Tmean* Average monthly mean air temperature; *GDP* Gross domestic product; *RH* Average monthly mean relative humidity; *Cropland* Annual average percentage of cropland; *Tmax* Average monthly maximum air temperature; *Impervious* Annual average percentage of impervious; *Water* Annual average percentage of water; *E–H region* The eastern region of the Hu Line; *S-QH region*: The southern region of the Q-H Line; *SHAP*: The Shapley Additive exPlanations

Among the influencing factors, imported cases contributed the most to local DF epidemics in the E–H (24.82%, Fig. 5a) and S-QH (31.01%, Fig. 5b) regions. Moreover, five variables associated with much greater contributions included Tmin (16.88%), Forest (8.58%), Pop (7.36%), Prec (7.28%), and Tmean (7.14%) in the E–H region, which differed from those in the S-QH region (GDP, Pop, RH, Cropland, and Forest). In addition, natural factors (i.e., five meteorological elements) were associated with greater contributions (41.04%) than socioeconomic factors (34.15%) in the E–H region. In comparison, socioeconomic factors were associated with greater contributions (44.26%) than natural conditions (24.74%) in the S-QH region.

**Fig. 6:**
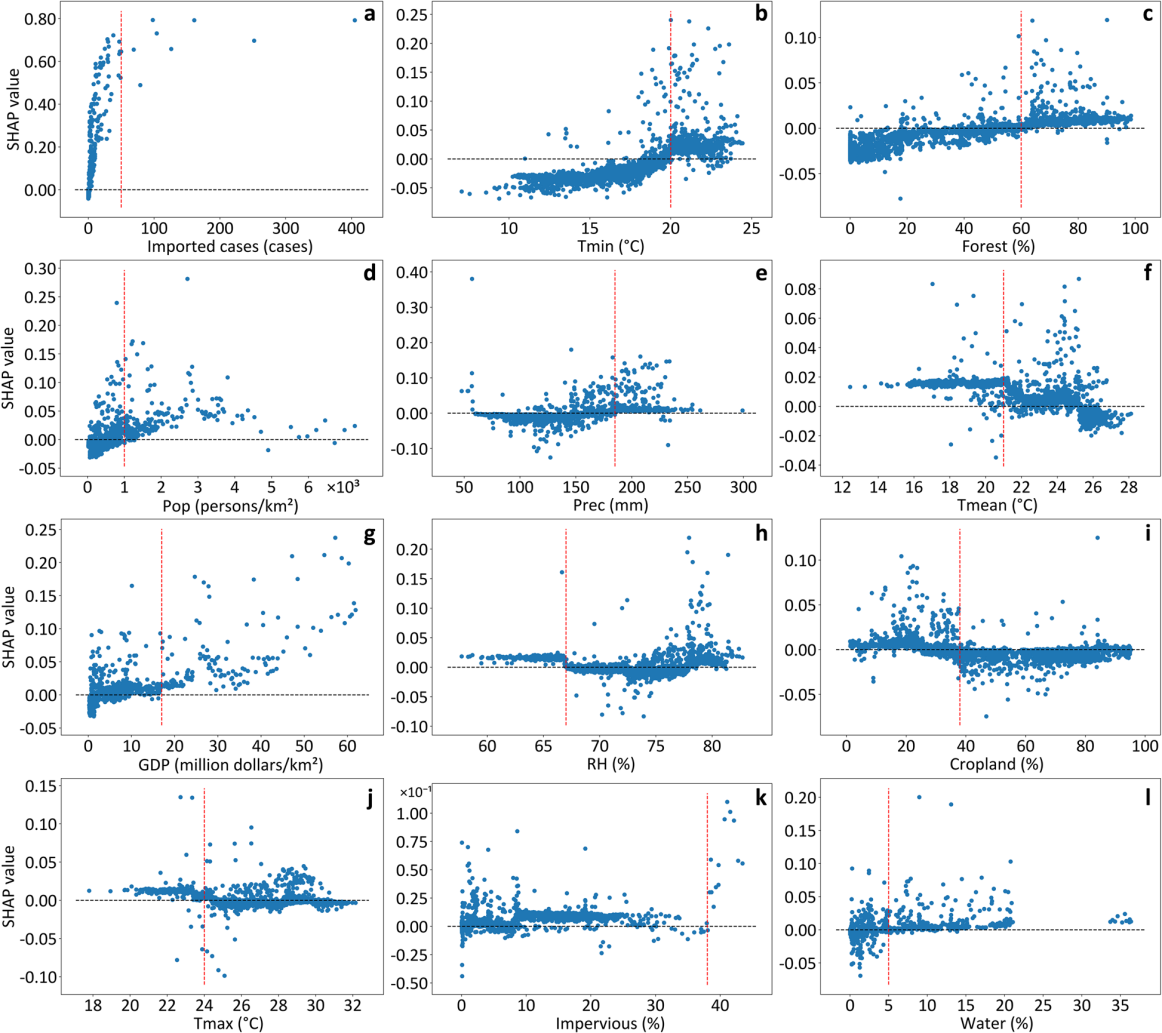
Relationships between 12 inputs and local dengue fever occurrence according to local SHAP values at the city level in the E–H region. **a** Number of imported cases (Imported cases); **b** Average monthly minimum air temperature (Tmin); **c** Annual average percentage of forest (Forest); **d** Population density (Pop); **e** Average monthly precipitation (Prec); **f** Average monthly mean air temperature (Tmean); **g** Gross domestic product (GDP); **h** Average monthly mean relative humidity (RH); **i** Annual average percentage of cropland (Cropland); **j** Average monthly maximum air temperature (Tmax); **k** Annual average percentage of impervious (Impervious); **l** Annual average percentage of water (Water). *E–H region* The eastern region of the Hu Line; *SHAP* The Shapley Additive exPlanations

According to the SHAP values derived from Model 1 (Fig. 6), the imported cases and the other 11 factors had complex impacts (protective or risk effects) on the occurrence of local DF epidemics at the city level. In the E–H region, the promoting effect of imported cases had a strengthening trend as the number of imported cases increased (Fig. 6a). In contrast, the other 11 factors generally presented composite promoting and inhibiting effects. These factors were categorized into two groups: Group 1 (i.e., Tmin, Forest, Pop, Prec, GDP, Impervious, and Water), which exhibited inhibiting effects before promoting effects, and Group 2 (i.e., Tmean, RH, Cropland, and Tmax), which exhibited promoting effects before inhibiting effects. Among the top five factors (Fig. 5a) derived from the contribution analyses, Tmin, Forest, Pop, and Prec belonged to Group 1, with respective inflection values of 20 °C (Fig. 6b), 60% (Fig. 6c), 1000 persons per square kilometer (Fig. 6d), and 185 millimeters (Fig. 6e). In contrast, Tmean belonged to Group 2, and its promoting effects became inhibitory at 21 °C (Fig. 6f). Similarly, the other six factors were categorized into Group 1 (GDP, Impervious, and Water) and Group 2 (RH, Cropland, and Tmax). In comparison, the factors in the S-QH region had similar composite impacts on the occurrence of local DF epidemics in this region (Additional file 1: Fig. S8), although their inflection values differed slightly from those in the E–H region. These analyses showed that the SHAP values were more reasonable than the Spearman correlation coefficients for interpreting the influences of potential factors on local epidemics. In addition, the fact that imported cases triggered local epidemics was further validated.

### Predicting the risk of local DF epidemics at the city level

Due to its good performance (AUC = 0.91), Model 1 was utilized to predict the occurrence probability of local DF epidemics at the city level in the E–H region. In this region, a total of 295 cities had various occurrence probabilities of local DF epidemics and were then categorized into five groups (Table 5). Among them, 20 cities in the first group (0.8 < probability < 1.0) were indeed affected by local DF epidemics in 2019, demonstrating the highest hitting ratio (100%). Furthermore, the hitting ratios of the other four groups (i.e., 0.6–1.0, 0.4–1.0, 0.2–1.0, and 0.0–1.0) gradually decreased to 95.56%, 79.41%, 67.65%, and 29.15%, respectively. In comparison, the hitting ratios of Model 1 clearly declined during 2020–2022, although only one city (Guangzhou) in 2020 had the highest probability of occurrence of local DF epidemics.

**Table 5:** The ability of Model 1 to predict the occurrence probability of local dengue fever epidemics at the city level in the E–H region

Year	Statistics of the cities	Probability of local DF epidemics				
		0.8–1.0	0.6–1.0	0.4–1.0	0.2–1.0	0.0–1.0
2019	Number of cities identified (NCI)	20	45	68	102	295
	Number of cities with actual DF epidemics (NCA)	20	43	54	69	86
	Hitting ratios	100.00%	95.56%	79.41%	67.65%	29.15%
2020	Number of cities identified (NCI)	1	2	5	23	295
	Number of cities with actual DF epidemics (NCA)	1	1	2	2	4
	Hitting ratios	100.00%	50.00%	40.00%	8.70%	1.36%
2021	Number of cities identified (NCI)	0	1	5	17	295
	Number of cities with actual DF epidemics (NCA)	0	0	0	0	1
	Hitting ratios	–	0.00%	0.00%	0.00%	0.34%
2022	Number of cities identified (NCI)	0	1	4	14	295
	Number of cities with actual DF epidemics (NCA)	0	0	1	1	2
	Hitting ratios	–	0.00%	25.00%	7.14%	0.68%

The probabilities, like 0.801.0, 0.60–0.80, 0.40–0.60, 0.20–0.40, and 0–0.20, were respectively categorized as the highest, high, moderate, low, and the lowest risk levels

The hitting ratio represents the percentage of NCA in NCI

*NCA* The number of cities with actual DF epidemics, *NCI* The number of cities identified, *DF* Dengue fever, *E–H region* The eastern region of the Hu Line

–Not applicable

**Fig. 7:**
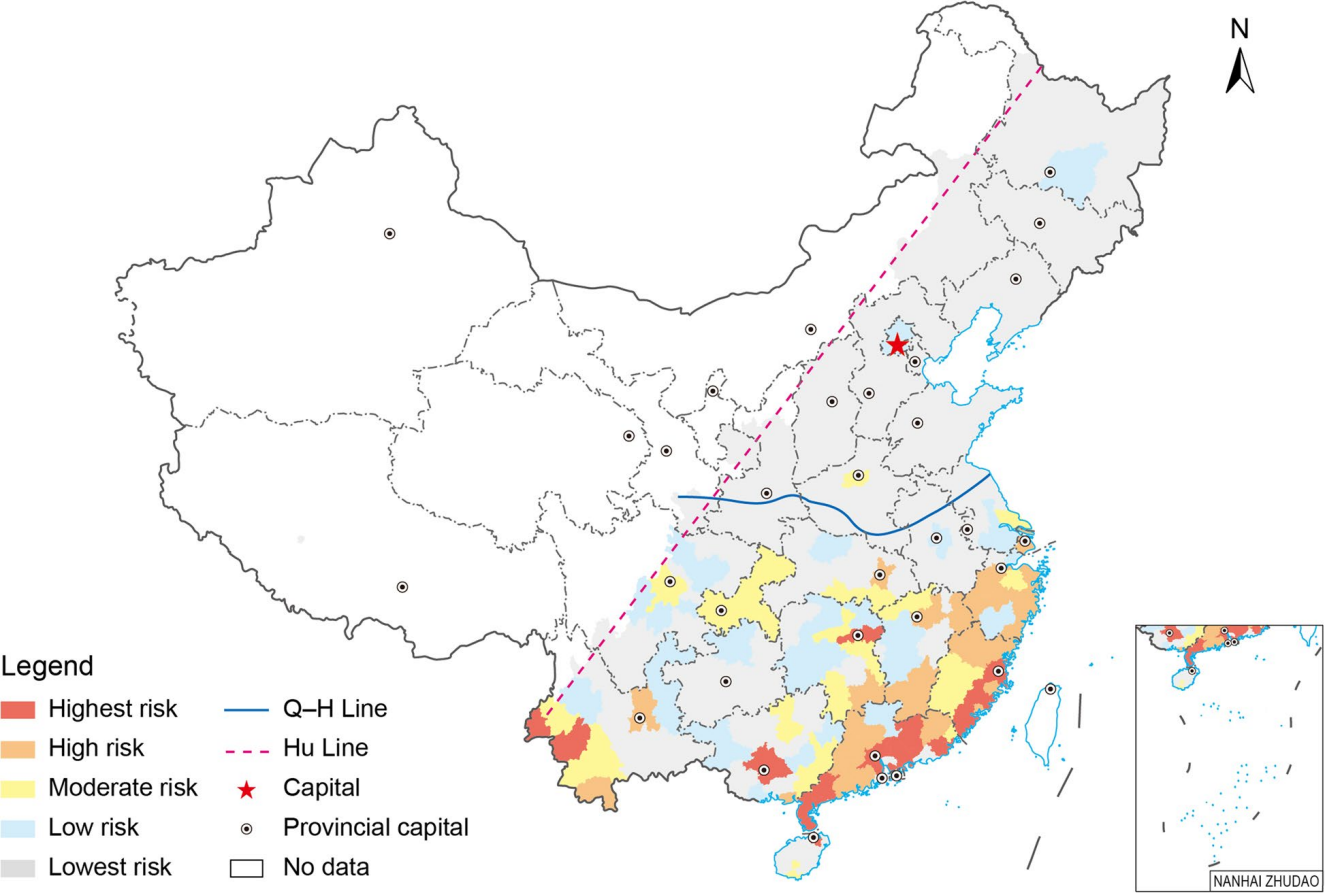
The city-level risk of local dengue fever epidemics in 2019 in China. *Hu Line* The Huhuanyong Line; *Q-H Line* The Qin Mountain-Huai River Line. Map approval No.: GS (2024) 2127

The cities at various risk levels for local DF epidemics displayed spatial disparities in the E–H region from 2019 to 2022. Most of the cities with the highest risk (20 cities) were located in the southeastern coastal areas (16 cities). Moreover, 25 high-risk and 23 moderate-risk cities were identified in the southeastern and central regions, respectively (Fig. 7). Thus, the above 68 cities were primarily concentrated in the S-QH region. During 2020–2022, the cities at various risk levels were also mainly located in this region (Additional file 1: Fig. S9). These results showed that the risk of local DF epidemics in the E–H region was appropriately predicted at the city level using RF models considering the time windows for local DF transmission.

## Discussion

Since DF epidemics in China have become increasingly severe in recent years, it is crucial to reveal the comprehensive features of DF outbreaks to accurately map the risk. Our study analyzed the spatiotemporal situation of China’s DF epidemic and identified potential influencing factors to map the risk of city-level local DF epidemics using RF models. Several notable findings were obtained and could provide valuable insight for developing targeted interventions for this disease.

Previous studies have demonstrated that DF epidemics pose an increasingly severe threat in China in terms of incidence rates or other indices of various spatial scales [7, 9, 16, 40] and that local epidemics closely correlated with imported epidemics in recent years [41, 42]. Similarly, our study revealed that DF epidemics tended to be unavoidable in China, especially in the E–H region. This finding can be explained by the following: First, imported epidemics seem to be inevitable in China given the increasingly large numbers of imported DF cases from endemic countries or territories (e.g., Southeast Asia and Central America) owing to a closer and stronger connection between China and these countries/territories in recent years [41, 43]. In addition to some traditional regions (i.e., Southeast and Southwest China) that are frequently infected by imported epidemics [5, 6], inland regional hub cities (e.g., provincial capitals) characterized by larger airports and export-oriented economies are constantly confronted with an increasing number of inbound or outbound tourists [44, 45]. Second, local DF epidemics were more easily triggered by imported cases due to their increasing ability to initiate local DF epidemics over time. Local DF epidemics in some cities (e.g., Guangzhou) were still caused by imported DF cases, even though the number of imported cases sharply decreased due to the strict immigration control and quarantine policies implemented in China during the COVID-19 pandemic in 2020–2022 [46]. Third, the spillover effect of DF (either imported or local cases) from traditional regions or inland hub cities to their surrounding cities is another reasonable explanation [9, 47], especially when susceptible cities with sustainable environmental conditions demonstrate opportune time windows for local DF transmission. Hence, it can be concluded that future DF epidemics seem to be inevitable in China. Accordingly, we cautiously suggest that more attention should be given to inland regional hub cities and their surrounding cities demonstrating time windows for local DF transmission, especially considering the ever-growing number of imported cases and their increasing initiating ability.

Moreover, the spatial distribution of city-level DF epidemics conformed to the geographical divisions of the Hu Line and Q-H Line in China. The city-level local DF epidemics were still geographically limited within the E–H region, especially in the S-QH region, which might be explained by the time windows for mosquito vector activity (Additional file 1: Fig. S6) since this disease was transmitted by *Aedes* species (*Aedes albopictus* and *Aedes aegypti*) in some specific phases with suitable environmental conditions [11, 12]. Moreover, the match degrees among the city-level occurrences of actual local epidemics and the time windows for mosquito vector activity or local DF transmission were satisfactory in the E–H region. Under these circumstances, it was reasonable that local DF epidemics occasionally occurred in cities in the N-QH region with late-beginning and short-term time windows after DF cases were imported from abroad or domestic regions. That is, time windows play a crucial and nonnegligible role in local DF epidemics in China. Thus, we believe that time windows provide helpful information for relevant departments to implement timely interventions for this disease.

Furthermore, the dominant influencing factors of city-level local DF epidemics differed within the E–H (i.e., the N-Q and S-QH regions), which might be partially attributable to the coefficient of variation (CV) of these factors (Additional file 1: Table S7). This finding was similar to that obtained in our earlier study of the dominant factors influencing DF epidemics in two traditional hotspot regions (the Pearl River Delta and the border area between Yunnan of China and Myanmar) [17]. Thus, it can be concluded that China’s DF epidemic is geographically restricted by time windows and spatially characterized by regionally distinct influencing factors. Therefore, we advise that health authorities in each city consider the status of time windows and regional attributes of potential influencing factors when planning or implementing efficient interventions for this disease.

In addition, our study revealed the inevitability and occasionality of China’s DF epidemic, which could improve our knowledge of the transmission and spread of this disease, especially in nonendemic areas such as China. Feasible solutions should be developed to address the challenges posed by the inevitability and occasionality of DF epidemics. First, it is crucial to monitor overseas DF epidemics and promptly acquire information about inbound tourists from endemic areas to assess the situation of imported DF epidemics. Second, sufficient surveillance of climatic elements should be efficiently utilized to confirm the status of time windows for local DF transmission, especially in inland regional hub cities and their surrounding areas. The final and key point is to construct a robust and reliable prediction model using RF models that health authorities can use to implement targeted measures to prevent and control DF. The two prerequisites to this end are as follows: Natural foci of this disease cannot be eliminated worldwide in the short term; and China remains a nonendemic region for DF.

Several limitations are worth noting. First, climatic data with a higher temporal resolution (e.g., weekly, ten-day) would be beneficial for more accurately characterizing the time windows at the city level; the match degree between the time windows and actual stages of local DF epidemics may increase, and effective interventions could be more precisely and timely implemented. Second, the effectiveness of city-level time windows could be further validated by obtaining synchronous surveillance data of mosquito vectors, which could further improve the ability of RF models to fit relationships between local epidemics and potential factors within these time windows and predict city-level risk for local DF epidemics. Finally, as the spillover of DF cases among domestic regions plays a critical role in local DF transmission, it is crucial to propose scientifically efficient solutions to characterize the network of relationships among domestic cities or regions regarding factors such as population flows, economic exchanges, and space–time distances.

## Conclusions

China is experiencing an increasing occurrence of sporadic local DF epidemics driven by an unavoidably higher frequency and intensity of imported DF epidemics. This research has improved our understanding of the severity of DF epidemics and their influencing factors in China or similar nonendemic countries/territories/regions, providing valuable insight for health authorities seeking to improve their intervention capabilities against this disease.

### Supplementary Information

Additional file1: Fig. S1 The distribution of China at provincial-level administrative divisions. Table S1 The list of the potential driving factors. Fig. S2 The distribution of socioeconomic and natural factors (a–k) in 2019 and geographical zoning (l) divided by Hu Line and Q-H Line. Table S2 Yearly numbers of total, imported, and local dengue fever cases in China during 2003–2022. Table S3 Yearly proportions of imported and local dengue fever cases to total cases, and the ratio of local cases to imported cases in China during 2003–2022. Table S4 Yearly numbers of cities with different dengue fever epidemics in China during 2003–2022. Table S5 Yearly proportions of cities with different dengue fever epidemics in the S-QH region during 2003–2022. Fig. S3 The distribution of the ratio of local dengue fever cases to imported dengue fever cases during 2003–2022. Fig. S4 The distribution of the cities with different dengue fever epidemics during 2003–2022. Fig. S5 Spatial distribution of the frequency and intensity of dengue fever epidemics during 2003–2022. Fig. S6 Time windows for city-level mosquito vector activity across China from 2003 to 2022. Fig. S7 Time windows for local dengue fever transmission across China from 2003 to 2022. Table S6 The AUC values derived from RF, GBM, and SVM models. Fig. S8 Relationships between 12 inputs and local dengue fever occurrence according to local SHAP values at the city level in the S-QH region. S1 Analyses of relationships between 12 inputs and local dengue fever occurrence in the S-QH region. Fig. S9 The city-level risk of local dengue fever epidemics in 2020 (a), 2021 (b), 2022 (c) in China. Table S7 The coefficient of variation of potential influencing factors in the E-H region, N-QH region, and S-QH region.

## Data Availability

The datasets used and analyzed during the current study are available from the corresponding author on reasonable request.

## Abbreviations

DF: Dengue fever
RF: Random forest
GBM: Gradient Boosting Machine
SVM: Support Vector Machine
ROC: Receiver operating characteristic
AUC: Area under the curve
SHAP: SHapley Additive exPlanations
Hu Line: Huhuanyong Line
Q-H Line: Qin Mountain-Huai River Line
W–H region: The western region of the Hu Line;
E–H region: The eastern region of the Hu Line;
N-QH region: The northern region of the Q-H Line;
S-QH region: The southern region of the Q-H Line;
NCI: Number of cities identified
NCA: Number of cities with actual dengue fever occurrences
CV: The coefficient of variation
COVID-19: Coronavirus disease 2019
