# Supplementary material for: Forecasting and mapping dengue fever epidemics in China: a spatiotemporal analysis
Source: Infect Dis Poverty. 2024 Jul 3;13:50. doi: 10.1186/s40249-024-01219-y (PMC11221048; doi:10.1186/s40249-024-01219-y)
Supplement: Supplementary file 1 — Additional file1: Fig. S1 The distribution of China at provincial-level administrative divisions. Table S1 The list of the potential driving factors. Fig. S2 The distribution of socioeconomic and natural factors (a–k) in 2019 and geographical zoning (l) divided by Hu Line and Q-H Line. Table S2 Yearly numbers of total, imported, and local dengue fever cases in China during 2003–2022. Table S3 Yearly proportions of imported and local dengue fever cases to total cases, and the ratio of local cases to imported cases in China during 2003–2022. Table S4 Yearly numbers of cities with different dengue fever epidemics in China during 2003–2022. Table S5 Yearly proportions of cities with different dengue fever epidemics in the S-QH region during 2003–2022. Fig. S3 The distribution of the ratio of local dengue fever cases to imported dengue fever cases during 2003–2022. Fig. S4 The distribution of the cities with different dengue fever epidemics during 2003–2022. Fig. S5 Spatial distribution of the frequency and intensity of dengue fever epidemics during 2003–2022. Fig. S6 Time windows for city-level mosquito vector activity across China from 2003 to 2022. Fig. S7 Time windows for local dengue fever transmission across China from 2003 to 2022. Table S6 The AUC values derived from RF, GBM, and SVM models. Fig. S8 Relationships between 12 inputs and local dengue fever occurrence according to local SHAP values at the city level in the S-QH region. S1 Analyses of relationships between 12 inputs and local dengue fever occurrence in the S-QH region. Fig. S9 The city-level risk of local dengue fever epidemics in 2020 (a), 2021 (b), 2022 (c) in China. Table S7 The coefficient of variation of potential influencing factors in the E-H region, N-QH region, and S-QH region. [file 40249_2024_1219_MOESM1_ESM.docx]

**Additional file 1**

Supplementary material to: Forecasting and mapping dengue fever epidemics in China: a spatiotemporal analysis.

**Table of Contents**

| **Page** | **Item** |
| --- | --- |
| 2 | Fig.S1 The distribution of China at provincial-level administrative divisions |
| 3 | Table S1 The list of the potential driving factors |
| 4 | Fig.S2 The distribution of socioeconomic and natural factors (a–k) in 2019 and geographical zoning (l) divided by Hu Line and Q-H Line |
| 5 | Table S2 Yearly numbers of total, imported, and local dengue fever cases in China during 2003–2022 |
| 5 | Table S3 Yearly proportions of imported and local dengue fever cases to total cases, and the ratio of local cases to imported cases in China during 2003–2022 |
| 6 | Table S4 Yearly numbers of cities with different dengue fever epidemics in China during 2003–2022 |
| 7 | Table S5 Yearly proportions of cities with different dengue fever epidemics in the S-QH region during 2003–2022 |
| 8 | Fig.S3 The distribution of the ratio of local dengue fever cases to imported dengue fever cases during 2003–2022 |
| 9 | Fig.S4 The distribution of the cities with different dengue fever epidemics during 2003–2022 |
| 10 | Fig.S5 Spatial distribution of the frequency and intensity of dengue fever epidemics during 2003–2022 |
| 11 | Fig.S6 Time windows for city-level mosquito vector activity across China from 2003 to 2022 |
| 12 | Fig.S7 Time windows for local dengue fever transmission across China from 2003 to 2022 |
| 12 | Table S6 The AUC values derived from RF, GBM, and SVM models |
| 13 | Fig.S8 Relationships between 12 inputs and local dengue fever occurrence according to local SHAP values at the city level in the S-QH region |
| 13 | S1 Analyses of relationships between 12 inputs and local dengue fever occurrence in the S-QH region |
| 14 | Fig.S9 The city-level risk of local dengue fever epidemics in 2020 (a), 2021 (b), 2022 (c) in China |
| 15 | Table S7 The coefficient of variation of potential influencing factors in the E-H region, N-QH region, and S-QH region |


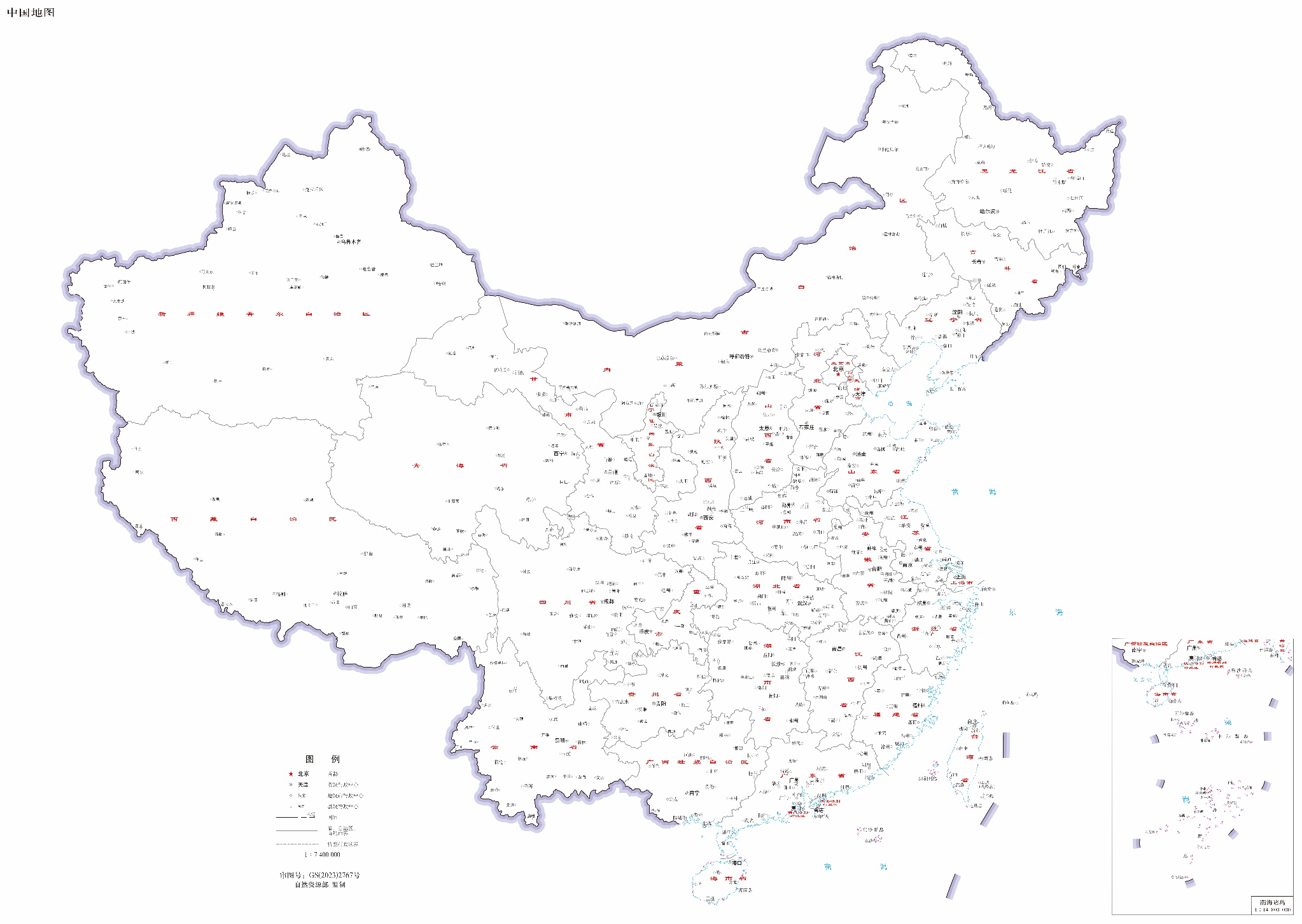


**Fig.S1 The distribution of China at provincial-level administrative divisions.**

(Source: <http://bzdt.ch.mnr.gov.cn/browse.html?picId=%224o28b0625501ad13015501ad2bfc2187%22>)

**Table S1 The list of the potential driving factors**

| **Short name** | **Long name** |
| --- | --- |
| Imported cases | Number of imported cases |
| GDP | Gross domestic product per capita |
| Pop | Population density |
| Cropland | Annual average percentage of cropland |
| Forest | Annual average percentage of forest |
| Water | Annual average percentage of water |
| Impervious | Annual average percentage of impervious |
| Tmax | Average monthly maximum air temperature |
| Tmean | Average monthly mean air temperature |
| Tmin | Average monthly minimum air temperature |
| RH | Average monthly mean relative humidity |
| Prec | Average monthly precipitation |


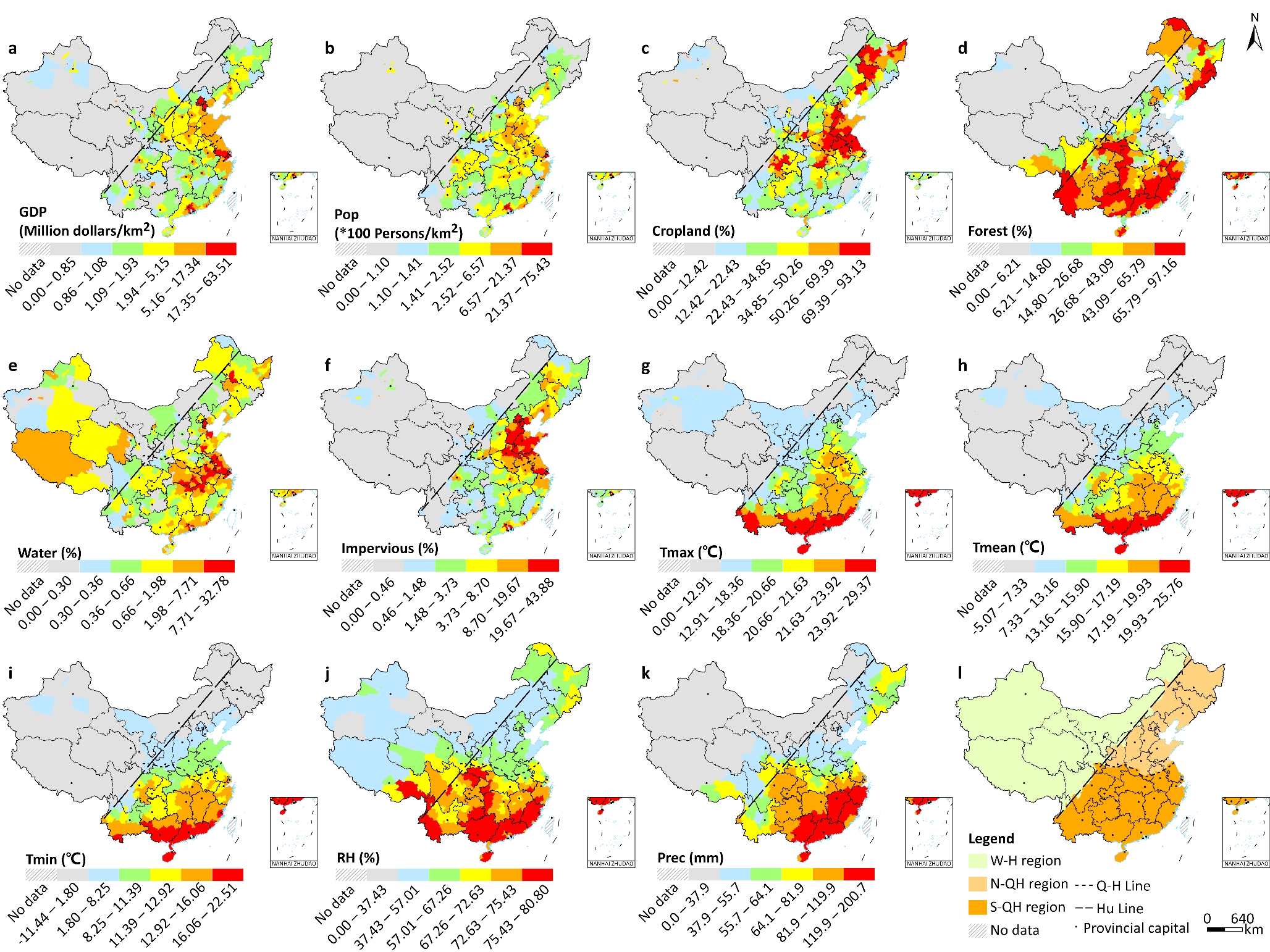


**Fig.S2 The distribution of socioeconomic and natural factors (a–k) in 2019 and geographical zoning (l) divided by Hu Line and Q-H Line.** GDP: Gross domestic product; Pop: Population density; Cropland: Annual average percentage of cropland; Forest: Annual average percentage of forest; Water: Annual average percentage of water; Impervious: Annual average percentage of impervious; Tmax: Average monthly maximum air temperature; Tmean: Average monthly mean air temperature; Tmin: Average monthly minimum air temperature; RH: Average monthly mean relative humidity; Prec: Average monthly precipitation. Hu Line: The Huhuanyong Line; Q-H Line: The Qin Mountain-Huai River Line. The E-H region: The eastern region of the Hu Line; The W-H region: The western region of the Hu Line; The S-QH region: The southern region of the Q-H Line; The N-QH region: The northern region of the Q-H Line.

**Table S2 Yearly numbers of total, imported, and local dengue fever cases in China during 2003–2022**

| **year** | **Total cases** | **Imported cases** | **Local cases** |
| --- | --- | --- | --- |
| 2003 | 76 | 5 | 71 |
| 2004 | 229 | 8 | 221 |
| 2005 | 37 | 37 | 0 |
| 2006 | 1044 | 36 | 1008 |
| 2007 | 532 | 46 | 486 |
| 2008 | 210 | 111 | 99 |
| 2009 | 294 | 63 | 231 |
| 2010 | 227 | 115 | 112 |
| 2011 | 140 | 100 | 40 |
| 2012 | 586 | 150 | 436 |
| 2013 | 4714 | 435 | 4279 |
| 2014 | 46975 | 484 | 46491 |
| 2015 | 4180 | 1089 | 3091 |
| 2016 | 2241 | 681 | 1560 |
| 2017 | 6840 | 2168 | 4672 |
| 2018 | 5536 | 1344 | 4192 |
| 2019 | 23290 | 5952 | 17338 |
| 2020 | 784 | 162 | 622 |
| 2021 | 37 | 28 | 9 |
| 2022 | 588 | 42 | 546 |

**Table S3 Yearly proportions of imported and local dengue fever cases to total cases, and the ratio of local cases to imported cases in China during 2003–2022**

| **year** | **Proportion of**  **imported cases (%)** | **Proportion of**  **local case (%)** | **Ratio of local to**  **imported cases (%)** |
| --- | --- | --- | --- |
| 2003 | 6.58 | 93.42 | 14.20 |
| 2004 | 3.49 | 96.51 | 27.63 |
| 2005 | 100.00 | 0.00 | 0.00 |
| 2006 | 3.45 | 96.55 | 28.00 |
| 2007 | 8.65 | 91.35 | 10.57 |
| 2008 | 52.86 | 47.14 | 0.89 |
| 2009 | 21.43 | 78.57 | 3.67 |
| 2010 | 50.66 | 49.34 | 0.97 |
| 2011 | 71.43 | 28.57 | 0.40 |
| 2012 | 25.60 | 74.40 | 2.91 |
| 2013 | 9.23 | 90.77 | 9.84 |
| 2014 | 1.03 | 98.97 | 96.06 |
| 2015 | 26.05 | 73.95 | 2.84 |
| 2016 | 30.39 | 69.61 | 2.29 |
| 2017 | 31.70 | 68.30 | 2.15 |
| 2018 | 24.28 | 75.72 | 3.12 |
| 2019 | 25.56 | 74.44 | 2.91 |
| 2020 | 20.66 | 79.34 | 3.84 |
| 2021 | 75.68 | 24.32 | 0.32 |
| 2022 | 7.14 | 92.86 | 13.00 |

**Table S4 Yearly numbers of cities with different dengue fever epidemics in China during 2003–2022**

| **year** | **Either imported or local epidemics** | **Both imported and local epidemics** | **Imported epidemics only** | **Local epidemics only** |
| --- | --- | --- | --- | --- |
| 2003 | 3 | 1 | 0 | 0 |
| 2004 | 9 | 1 | 0 | 0 |
| 2005 | 16 | 0 | 3 | 0 |
| 2006 | 18 | 2 | 4 | 0 |
| 2007 | 22 | 2 | 3 | 1 |
| 2008 | 32 | 5 | 6 | 0 |
| 2009 | 20 | 3 | 3 | 0 |
| 2010 | 32 | 4 | 7 | 0 |
| 2011 | 27 | 2 | 5 | 0 |
| 2012 | 38 | 2 | 11 | 0 |
| 2013 | 64 | 9 | 22 | 1 |
| 2014 | 88 | 31 | 21 | 9 |
| 2015 | 90 | 16 | 26 | 0 |
| 2016 | 87 | 12 | 35 | 1 |
| 2017 | 111 | 21 | 47 | 0 |
| 2018 | 141 | 25 | 68 | 0 |
| 2019 | 219 | 83 | 127 | 3 |
| 2020 | 51 | 3 | 17 | 0 |
| 2021 | 8 | 1 | 1 | 0 |
| 2022 | 13 | 2 | 0 | 0 |

**Table S5 Yearly proportions of cities with different dengue fever epidemics in the S-QH region during 2003–2022**

| **year** | **Either imported or local epidemics (%)** | **Both imported and local epidemics (%)** | **Imported epidemics only (%)** | **Local epidemics only (%)** |
| --- | --- | --- | --- | --- |
| 2003 | 100.00 | 100.00 | 0.00 | 0.00 |
| 2004 | 100.00 | 100.00 | 0.00 | 0.00 |
| 2005 | 87.50 | 0.00 | 66.67 | 0.00 |
| 2006 | 77.78 | 100.00 | 25.00 | 0.00 |
| 2007 | 90.91 | 100.00 | 100.00 | 0.00 |
| 2008 | 87.50 | 100.00 | 66.67 | 0.00 |
| 2009 | 95.00 | 100.00 | 100.00 | 0.00 |
| 2010 | 90.63 | 100.00 | 71.43 | 0.00 |
| 2011 | 88.89 | 100.00 | 60.00 | 0.00 |
| 2012 | 76.32 | 100.00 | 45.45 | 0.00 |
| 2013 | 78.13 | 100.00 | 50.00 | 0.00 |
| 2014 | 84.09 | 100.00 | 42.86 | 88.89 |
| 2015 | 83.33 | 100.00 | 57.69 | 0.00 |
| 2016 | 75.86 | 100.00 | 48.57 | 100.00 |
| 2017 | 79.28 | 95.24 | 57.45 | 0.00 |
| 2018 | 77.30 | 100.00 | 58.82 | 0.00 |
| 2019 | 72.15 | 95.18 | 55.91 | 100.00 |
| 2020 | 84.31 | 100.00 | 70.59 | 0.00 |
| 2021 | 87.50 | 100.00 | 0.00 | 0.00 |
| 2022 | 92.31 | 100.00 | 0.00 | 0.00 |
| Note: The S-QH region: The southern region of the Q-H Line. | | | | |


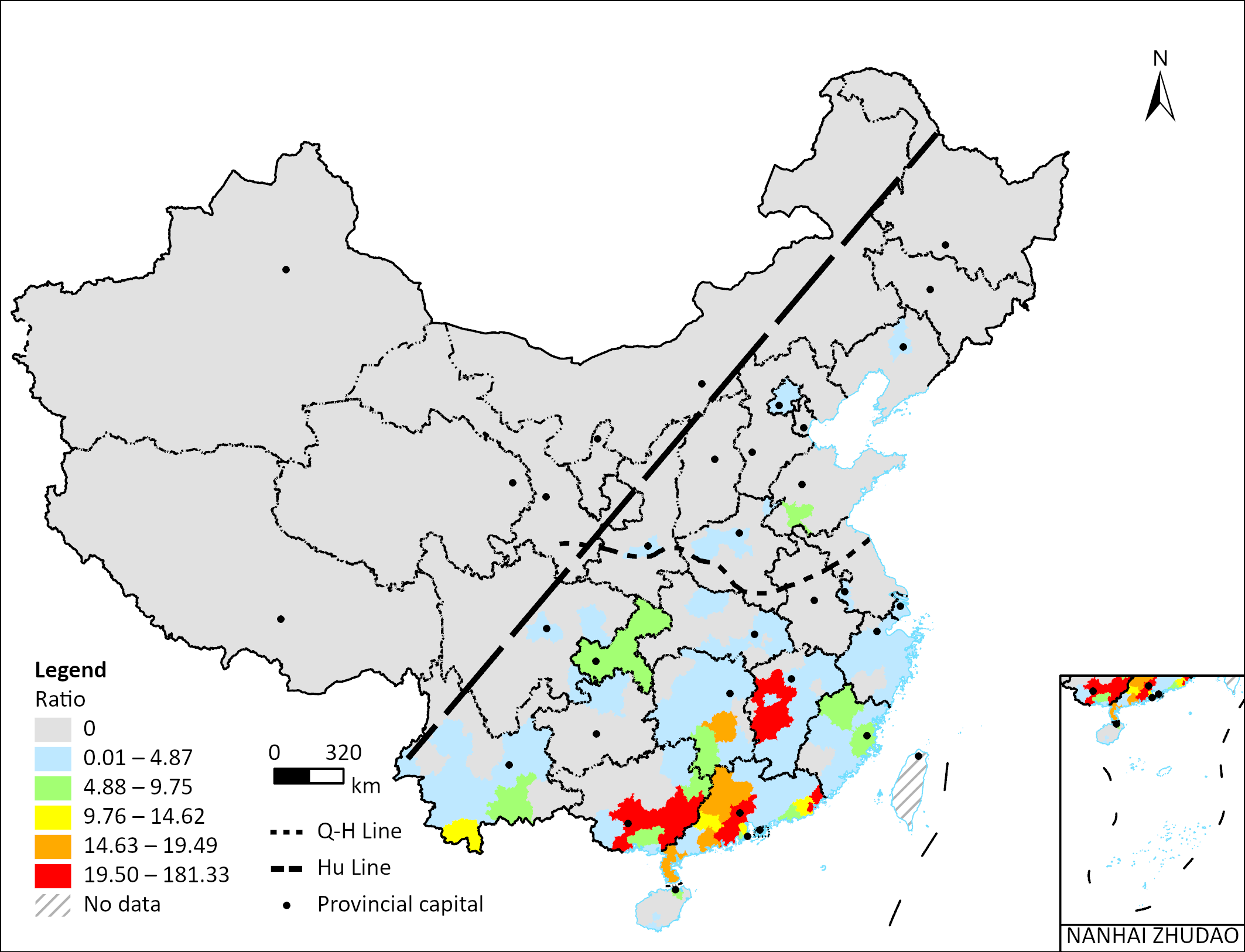


**Fig.S3 The distribution of the ratio of local dengue fever cases to imported dengue fever cases during 2003–2022.** Hu Line: The Huhuanyong Line; Q-H Line: The Qin Mountain-Huai River Line.


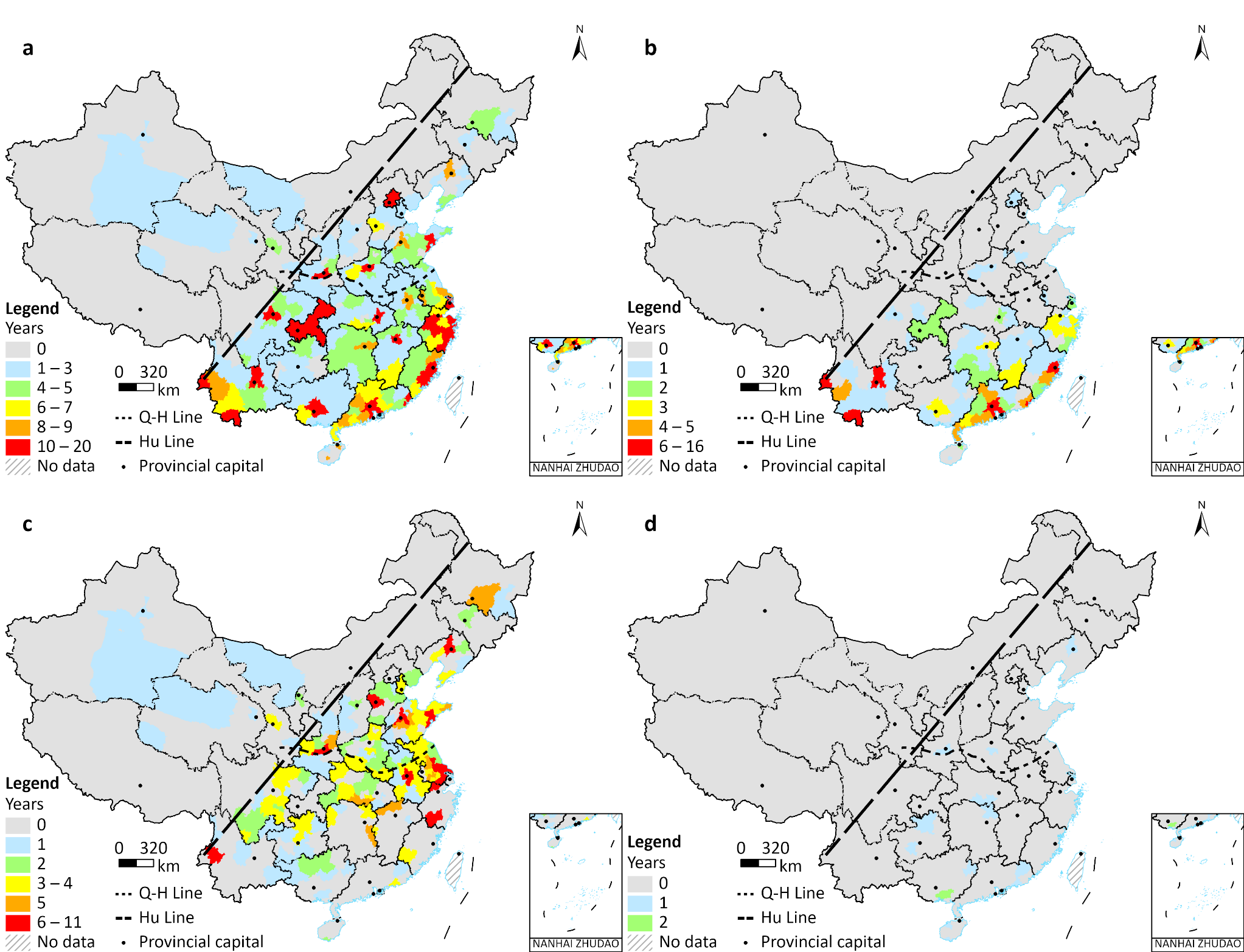


**Fig.S4 The distribution of the cities with different dengue fever epidemics during 2003–2022.** (a) Cities with either imported or local DF epidemics; (b) Cities with both imported and local DF epidemics; (c) Cities with only imported DF epidemics; (d) Cities with only local DF epidemics. DF: Dengue fever; Hu Line: The Huhuanyong Line; Q-H Line: The Qin Mountain-Huai River Line. Map approval No.: GS (2023) 2767.


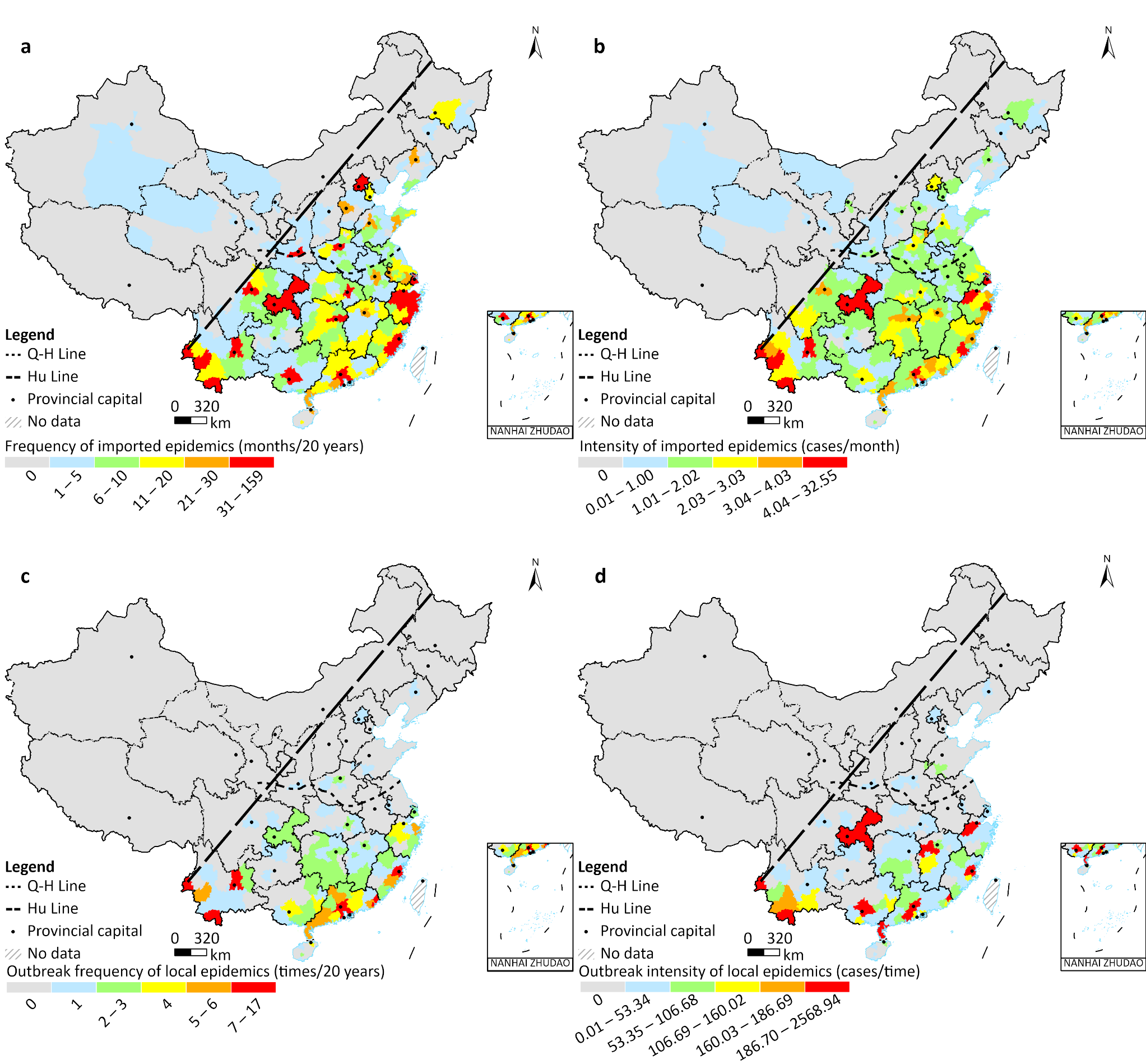


**Fig.S5 Spatial distribution of the frequency and intensity of dengue fever epidemics during 2003–2022.** (a) The frequency of imported DF epidemics; (b) The intensity of imported DF epidemics; (c) The distribution of the frequency of local DF outbreaks; (d) The distribution of the intensity of local DF outbreaks. DF: Dengue fever; Hu Line: The Huhuanyong Line; Q-H Line: The Qin Mountain-Huai River Line. Map approval No.: GS (2023) 2767.


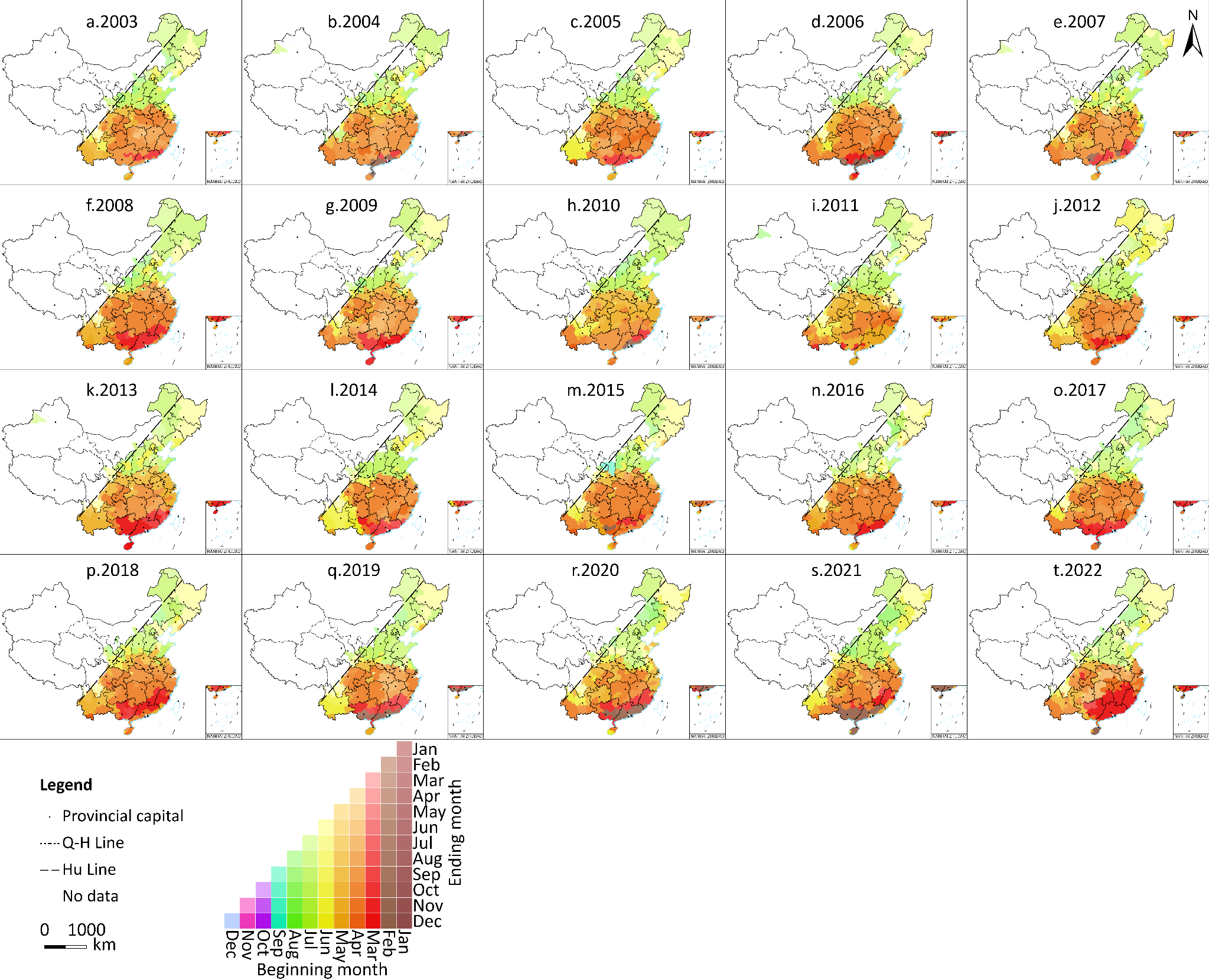


**Fig.S6 Time windows for city-level mosquito vector activity across China from 2003 to 2022.** (a–t) Yearly time windows for mosquito vector activity from 2003 to 2022. The S-QH region: The southern region of the Q-H Line; The N-QH region: The northern region of the Q-H Line; Hu Line: The Huhuanyong Line; Q-H Line: The Qin Mountain-Huai River Line. Map approval No.: GS (2023) 2767.


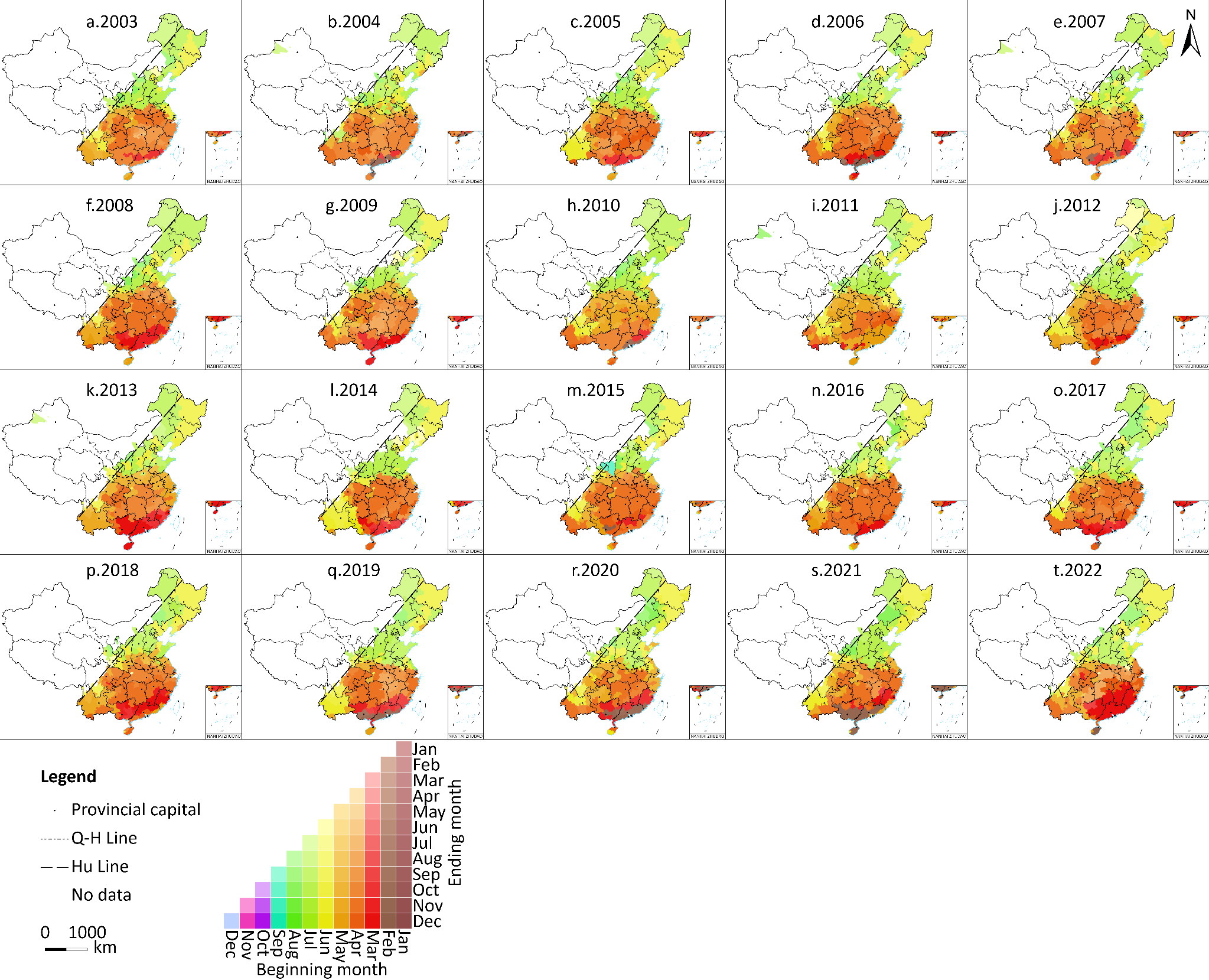


**Fig.S7 Time windows for** **local dengue fever transmission across China from 2003 to 2022.** (a–t) Yearly time windows for local dengue fever transmission from 2003 to 2022. The S-QH region: The southern region of the Q-H Line; The N-QH region: The northern region of the Q-H Line; Hu Line: The Huhuanyong Line; Q-H Line: The Qin Mountain-Huai River Line. Map approval No.: GS (2023) 2767.

**Table S6 The AUC values derived from RF, GBM, and SVM models**

|  | The E-H region | |  | The S-QH region | |
| --- | --- | --- | --- | --- | --- |
|  | Training | Testing |  | Training | Testing |
| RF | 0.87 | 0.92 |  | 0.88 | 0.85 |
| GBM | 0.88 | 0.90 |  | 0.87 | 0.87 |
| SVM | 0.80 | 0.79 |  | 0.80 | 0.75 |
| **Note**: AUC: Area Under the Curve; RF: Random forest; GBM: Gradient boosting machine; SVM: Support vector machine. The E-H region: The eastern region of the Hu Line; The S-QH region: The southern region of the Q-H Line. | | | | | |


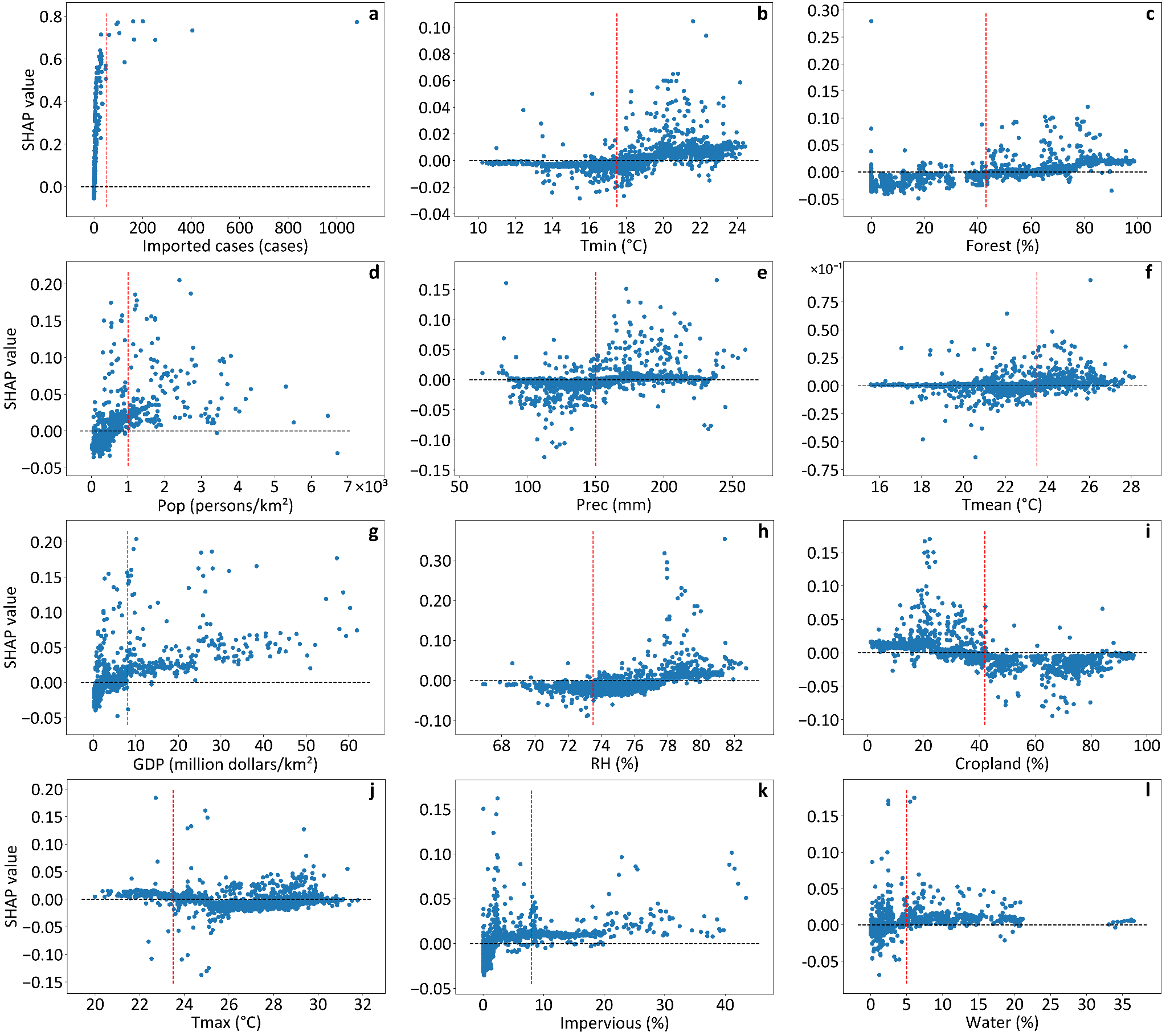


**Fig.S8 Relationships between 12 inputs and local dengue fever occurrence according to local SHAP values at the city level in the S-QH region. a** Number of imported cases (Imported cases); **b** Average monthly minimum air temperature (Tmin); **c** Annual average percentage of forest (Forest); **d** Population density (Pop); **e** Average monthly precipitation (Prec); **f** Average monthly mean air temperature (Tmean); **g** Gross domestic product (GDP); **h** Average monthly mean relative humidity (RH); **i** Annual average percentage of cropland (Cropland); **j** Average monthly maximum air temperature (Tmax); **k** Annual average percentage of impervious (Impervious); **l** Annual average percentage of water (Water). The S-QH region: The southern region of the Q-H Line; SHAP: The Shapley Additive exPlanations.

**S1 Analyses of relationships between 12 inputs and local dengue fever occurrence in the S-QH region.** According to the local SHAP values from Model 3 (**Fig.S8**), the promoting effects of imported cases increased rapidly as the number of imported cases increased (**Fig.S8**a), which was similar to the result in the E-H region. The other 11 factors generally presented composite promoting and inhibiting effects. These factors were categorized into two groups: Group 1 (i.e., GDP, Pop, RH, Forest, Imperious, Prec, Water, and Tmin), which exhibited inhibiting effects before promoting effects, and Group 2 (i.e., Cropland, Tmax and Tmean), which exhibited promoting effects before inhibiting effects. Among the top five factors derived from the contribution analyses, GDP, Pop, RH, and Forest belonged to Group 1, with respective inflection values of 8 million dollars per square kilometer (**Fig.S8**g), 1000 persons per square kilometer (**Fig.S8**d), 73.5% (**Fig.S8**h) and 43% (**Fig.S8**c). In contrast, Cropland belonged to Group 2, and its promoting effects became inhibitory at 42% (**Fig.S8**i). The other six factors were categorized into Group 1 (Imperious, Prec, Water, and Tmin) and Group 2 (Tmax and Tmean), respectively. Imperious, Prec, Water, and Tmin in Group 1 possessed respective inflection values of 8% (**Fig.S8**k), 150 millimeters (**Fig.S8**e), 5% (**Fig.S8**l), and 17.5 °C (**Fig.S8**b) while Tmax and Tmean in Group 2 both changed at the inflection value of 23.5 °C (**Fig.S8**j and **Fig.S8**f).


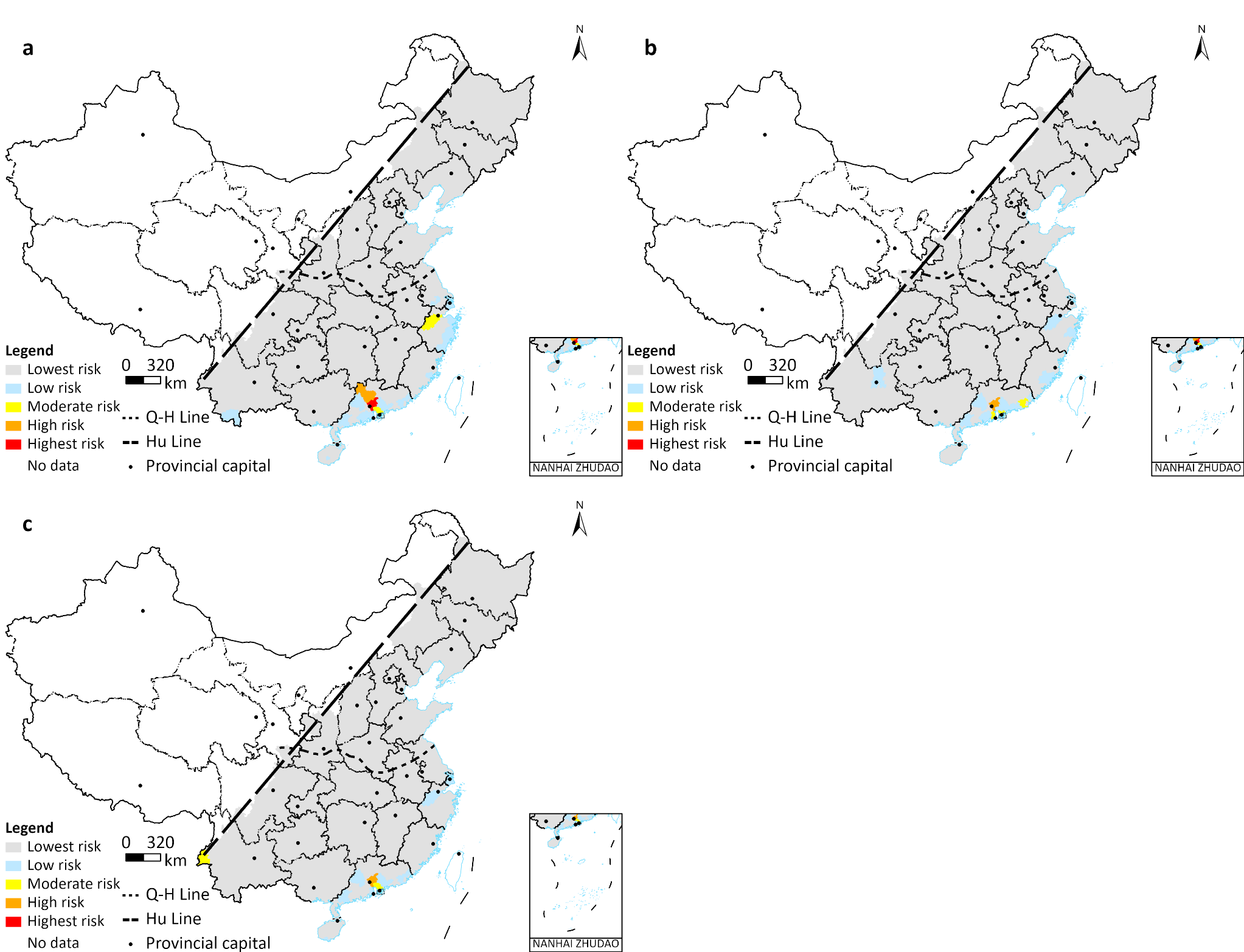
**Fig.S9 The city-level risk of local dengue fever epidemics in 2020 (a), 2021 (b), 2022 (c) in China.** Hu Line: The Huhuanyong Line; Q-H Line: The Qin Mountain-Huai River Line. Map approval No.: GS (2023) 2767.

**Table S7 The coefficient of variation of potential influencing factors in the E-H region, N-QH region, and S-QH region**

|  | **CV1** | **CV2** | **CV3** |
| --- | --- | --- | --- |
| Imported cases | 10.03 | 7.10 | 8.29 |
| GDP | 1.65 | 0.83 | 1.90 |
| Pop | 1.17 | 0.67 | 1.33 |
| Cropland | 0.53 | 0.38 | 0.59 |
| Forest | 0.73 | 1.09 | 0.53 |
| Water | 1.47 | 1.36 | 1.37 |
| Impervious | 1.11 | 0.68 | 1.46 |
| Tmax | 0.09 | 0.10 | 0.08 |
| Tmean | 0.11 | 0.12 | 0.10 |
| Tmin | 0.17 | 0.17 | 0.14 |
| RH | 0.06 | 0.06 | 0.03 |
| Prec | 0.27 | 0.24 | 0.22 |
| Note: CV: The coefficient of variation. CV1, CV2, and CV3 are the coefficient of variations in the E-H region, N-QH region, and S-QH region. Imported cases: Number of imported cases; GDP: Gross domestic product; Pop: Population density; Cropland: Annual average percentage of cropland; Forest: Annual average percentage of forest; Water: Annual average percentage of water; Impervious: Annual average percentage of impervious; Tmax: Average monthly maximum air temperature; Tmean: Average monthly mean air temperature; Tmin: Average monthly minimum air temperature; RH: Average monthly mean relative humidity; Prec: Average monthly precipitation. The E-H region: The eastern region of the Hu Line; The N-QH region: The northern region of the Q-H Line; The S-QH region: The southern region of the Q-H Line. | | | |
